# A Tailored Artificial Biocatalyst for Bacterial Endophthalmitis Therapy via Enhanced Ferroptosis‐Like Death

**DOI:** 10.1002/advs.202504601

**Published:** 2025-06-10

**Authors:** Caixia Sun, Yingying Jiang, Shibo Zhang, Zhipeng Ding, Lijun Pu, Xinmei Liu, Jun Yang, Xiangkai Zhuge, Jianjun Dai, Yanmin Ju

**Affiliations:** ^1^ College of Pharmacy China Pharmaceutical University Nanjing 211198 China; ^2^ Department of Ophthalmology The Affiliated Zhangjiagang Hospital of Soochow University Suzhou 215600 China; ^3^ Nanjing Institute for Food and Drug Control Nanjing 210038 China; ^4^ School of Public Health Nantong University Nantong 226019 China; ^5^ MOE Joint International Research Laboratory of Animal Health and Food Safety Key Laboratory of Animal Bacteriology Ministry of Agriculture College of Veterinary Medicine Nanjing Agricultural University Nanjing 210095 China

**Keywords:** artificial biocatalysts, bacterial endophthalmitis, enhanced ferroptosis‐like death, peptidoglycan targeting

## Abstract

Bacterial endophthalmitis is an ophthalmological emergency that can lead to permanent blindness, and high‐efficiency therapeutic strategies that can completely eradicate pathogens within a short timeframe are needed. However, intrinsic limitations of this disease, such as low administration frequency and dosage, render most currently available nano‐antibacterial strategies inapplicable. To address this challenge, a bio‐targeted catalytic strategy that is based on a bacteria‐specific artificial biocatalyst (MoS_2_/Fe@mercaptophenylboronic acid@hyaluronic acid, MFBH) and suitable for treating bacterial endophthalmitis is proposed. The results show that MFBH exhibits high‐efficiency peptidoglycan‐targeted catalytic antibacterial capacity against both standard and clinically isolated strains of *Staphylococcus aureus*. Notably, the in vivo results demonstrate that MFBH achieves effective treatment of bacterial endophthalmitis at an extremely low dose (≈4 µg kg^−1^) via a single intravitreal injection without causing retinal damage. Importantly, the therapeutic efficacy of MFBH is comparable to that of vancomycin. Mechanistic analysis reveals that MFBH induces enhanced ferroptosis‐like bacterial killing by accelerating reactive oxygen species (ROS) burst. Further investigations show that the generation of abundant ROS is closely associated with the sulfur vacancies and exposed of reactive Mo^4+^ on the surface of the prepared artificial biocatalyst. In summary, this bacteria‐specific artificial biocatalyst provides a promising strategy for treating endophthalmitis.

## Graphical abstract

A tailored bio‐targeted catalytic strategy based on a bacteria‐specific artificial biocatalyst (MoS_2_/Fe@mercaptophenylboronic acid@hyaluronic acid, MFBH) is proposed for treating bacterial endophthalmitis. This strategy exhibits high‐efficiency peptidoglycan‐targeted bactericidal efficacy in vivo following a single intravitreal injection of an extremely low dose of MFBH (≈4 µg kg^−1^). The mechanism is enhanced ferroptosis‐like bacterial death provoked by expediting reactive oxygen species burst.

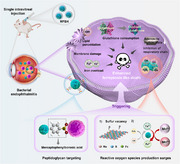

## 1 Introduction

Bacterial endophthalmitis is a devastating ocular medical emergency that can results in blindness. It is caused primarily by *Staphylococcus* infection and affects at least 9.5 million people worldwide.^[^ ^1^ ^]^ Irreversible visual impairment and even eye removal are common in patients who do not receive timely and effective treatment.^[^ ^2^ ^]^ Empirical intravitreal injection of vancomycin (VAN) is the first‐line treatment before the causative pathogen is identified, but this therapeutic strategy significantly increases the risk of bacterial resistance and retinal damage.^[^ ^3^ ^]^ Currently, alternative therapies to antibiotic treatment based on nanotechnology have emerged as compelling approaches for treating bacterial infectious diseases, and encouraging results have been obtained.^[^ ^4^ ^]^ However, these therapies are mostly unsuitable for treating bacterial endophthalmitis owing to the unique characteristics of the disease, including its acute onset and rapid progression, as well as its special ocular structures.^[^ ^2^, ^5^ ^]^ The use of low doses and infrequent administration via intravitreal injection is crucial, giventhe relatively enclosed structure of the eye and the strong susceptibility of photoreceptors and other retinal cells to stimuli.^[^ ^6^ ^]^ The administration of these alternative therapies at high doses and with high frequencies of intravitreal injection to eradicate bacteria can result in retinal damage and increased risk of intraocular infection. Although nanotechnologies, such as phototherapy, ion therapy, and hydrogel‐based therapies, have been explored for the treatment of bacterial endophthalmitis, the introduction of external light sources, precious metal ions, or chemical compounds often compromises treatment safety.^[^ ^5^, ^7^ ^]^ Thus, developing a novel antibacterial therapeutic strategy that is safe, highly effective and suitable for the treatment of endophthalmitis is urgently needed.

Iron‐based nanoagents, which are composed of biocompatible iron, efficiently eliminate pathogens by triggering bacterial ferroptosis‐like death, and their therapeutic efficiency can be improved by increasing their dosage owing to Fe^2+^‐dependent antibacterial activity.^[^ ^8^ ^]^ Nevertheless, the vulnerability of ocular tissues highlights the impracticality of this strategy in treating bacterial endophthalmitis. As previously reported, the ferroptosis‐like death can be initiated by reactive oxygen species (ROS)‐mediated lipid peroxidation.^[^ ^8^, ^9^ ^]^ Accordingly, to achieve satisfactory therapeutic efficacy at low doses, significantly improving the Fenton catalytic activity of iron‐based nanoagents to generate large amounts of ROS is essential. Molybdenum ions, which are key components of human molybdenum supplements, have been identified as co‐catalysts in Fenton chemistry, and they are capable of promoting ROS production.^[^ ^10^ ^]^ Thus, we envision that iron‐based nanoagents assisted by molybdenum ions can trigger robust bacterial ferroptosis‐like death through accelerating ROS burst, potentially achieving satisfactory therapeutic efficacy. Notably, ROS exhibit short lifetimes (shorter than 200 ns) and diffusion distances (≈20 nm).^[^ ^11^ ^]^ Only a tiny percentage of the ROS generated by untargeted iron‐based nanoagents can effectively act on bacteria due to the continuous intraocular movement of pathogens and nanoagents caused by eye rotation, which reduces the bactericidal efficacy of iron‐based nanoagents.^[^ ^12^ ^]^

**Scheme 1:**
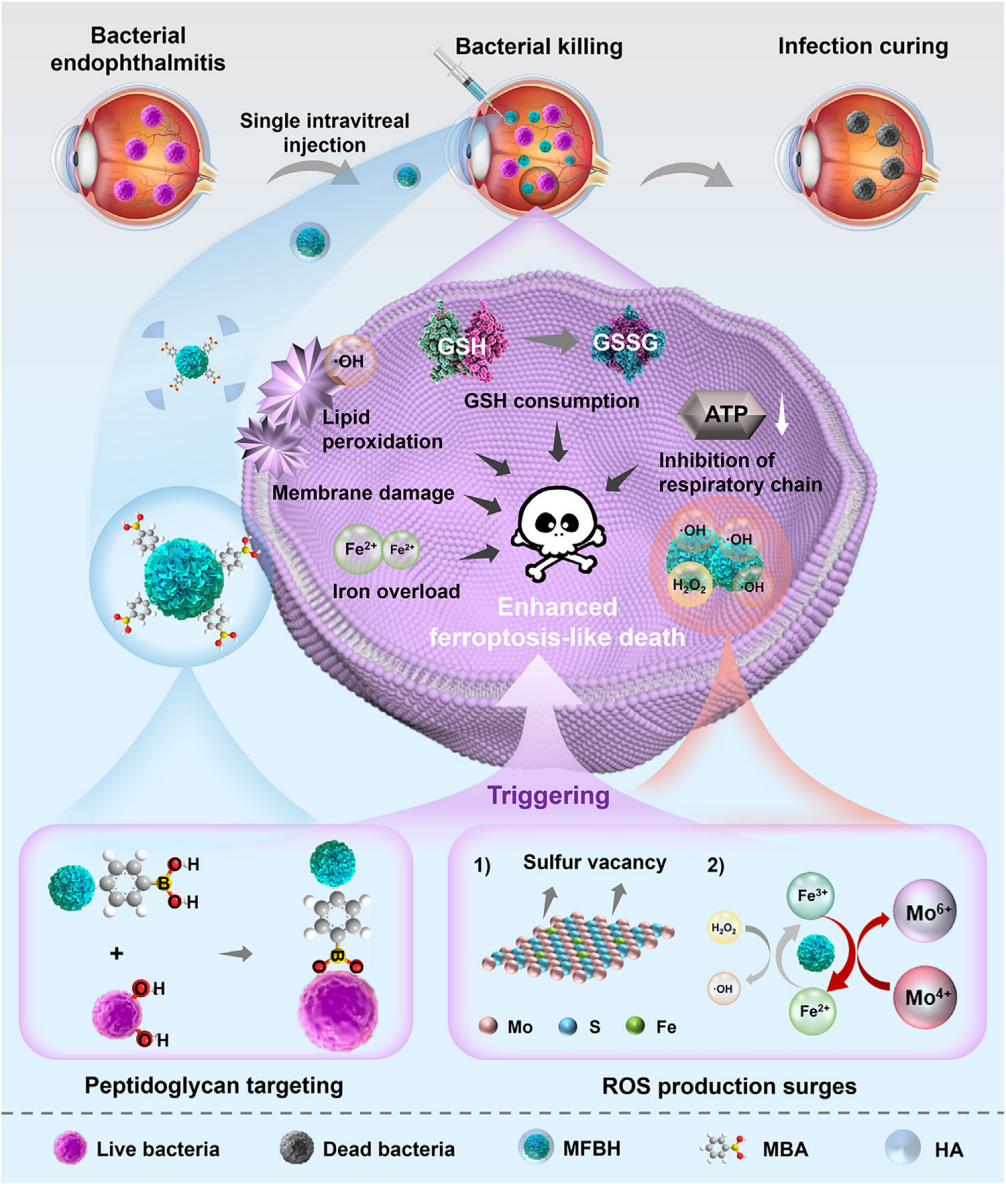
Schematic illustration of MFBH for bacterial endophthalmitis therapy. A low dose of MFBH following a single intravitreal injection achieves high‐efficiency peptidoglycan‐targeted bactericidal efficacy. The antibacterial mechanism is enhanced bacterial ferroptosis‐like death triggered mainly by expediting ROS burst.

Herein, we developed a bio‐targeted catalytic strategy based on a bacteria‐specific artificial biocatalyst (MoS_2_/Fe@mercaptophenylboronic acid@hyaluronic acid, MFBH) that is suitable for treating bacterial endophthalmitis (**Scheme** **1**). Briefly, MFBH was fabricated by modifying mercaptophenylboronic acid (MBA) and hyaluronic acid (HA) onto the surface of MoS_2_/Fe (MF) nanoflowers synthesized via a one‐pot solvothermal approach. We found that MFBH targeted bacterial peptidoglycans through MBA and exerted high‐efficiency catalytic antibacterial effects on both the ATCC 25 923 standard strain and clinically isolated strains of *Staphylococcus aureus* (*S. aureus*). Importantly, the in vivo results demonstrated that MFBH effectively treated bacterial endophthalmitis following a single intravitreal injection at an extremely low dose (≈4 µg kg^−1^) and presented comparable therapeutic efficacy to VAN without inducing retinal toxicity. The results of transcriptome sequencing (RNA‐seq) revealed that MFBH upregulated the expression of genes related to iron uptake, oxidative stress, and glutathione (GSH) consumption while downregulating the expression of genes associated with energy metabolism, indicating bacterial ferroptosis‐like death. Experiments investigating hallmarks of ferroptosis‐like death further confirmed this antibacterial mechanism. Notably, excess hydroxyl radicals (·OH) were the initiator for ferroptosis‐like bacterial killing, which primarily stems from the increased ROS generation capacity associated with sulfur vacancies and exposed reactive Mo^4+^ on the surface of MFBH. Attractively, compared with antibiotics, MFBH inhibited the development of bacterial resistance. Together, these results demonstrate the potential of this artificial biocatalyst as an effective nanodrug for the clinical treatment of bacterial endophthalmitis, expanding the clinical applications of biocatalysts.

## 2 Results and Discussion

### 2.1 Synthesis and Characterization of MFBH

**Figure 1:**
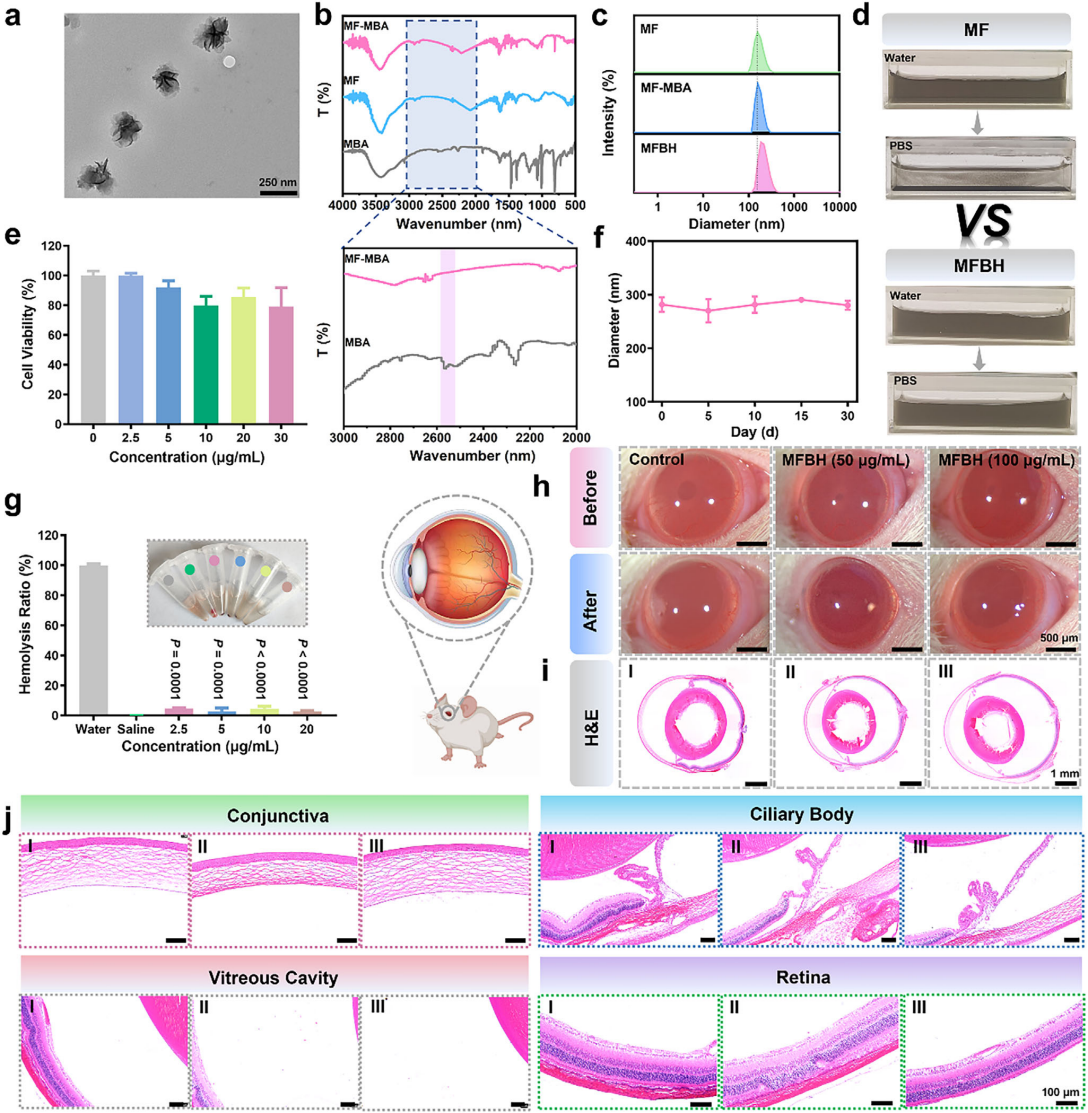
Characterizations of MFBH. a) Transmission electron microscopy images of MF nanoflowers. b) FTIR spectrum of MBA, MF nanoflowers and MF‐MBA. c) Dynamic light scattering (DLS) of MF nanoflowers, MF‐MBA and MFBH. d) Stability test of MF and MFBH in different systems. e) Cytocompatibility evaluation of MFBH. f) The DLS change curves of MFBH after being stored at room temperature for varying durations. g) Hemocompatibility assessment of MFBH. h) Representative ocular photographs captured on day 7 following a single intravitreal injection of different concentrations of MFBH, showing anterior segment morphology before and after treatment with compound tropicamide eye drops. i) Representative H&E staining images of ocular tissues collected on day 7 post a single intravitreal injection of different concentrations of MFBH. j) Representative H&E staining images of conjunctiva, ciliary body, vitreous cavity and retina collected on day 7 following single intravitreal injection of different concentrations of MFBH. I: Control, II: MFBH (100 µg mL^−1^), III: MFBH (200 µg mL^−1^). (Data are presented as mean ± SD. Statistical significance was assessed using Student's *t*‐tests. *n* = 3.).

The process of MFBH preparation is illustrated in Figure S1 (Supporting Information). As shown in **Figure** **1**a, the obtained MF nanoflowers were monodispersed and exhibited a flower‐like morphology with an approximate size of 200 nm. X‐ray diffraction results revealed that the (002) crystal plane of the MF nanoflowers shifted leftward from 14.2° to 9.2°, demonstrating the structural transformation from the 2H phase to the 1T phase as well as the successful incorporation of iron atoms (Figure S2, Supporting Information).^[^ ^13^ ^]^ Further validation was provided by Raman spectroscopy, which revealed the J_1_ (Mo‐Mo stretching vibrations), J_2_, E_1_ _g_ (octahedral coordination geometry of Mo), and J_3_ phonon modes specific to metallic 1T‐MoS_2_ (Figure S3, Supporting Information).^[^ ^14^ ^]^ The presence of Fe, Mo and S was indicated by X‐ray photoelectron spectroscopy (XPS) (Figure S4a, Supporting Information). The characteristic peaks of Fe 2p_3/2_ and Fe 2p_1/2_ were observed at 708.1 and 721.2 eV, respectively (Figure S4b, Supporting Information). The XPS peaks of S 2p_3/2_ and S 2p_1/2_ were observed at 161.6 and 162.8 eV, respectively (Figure S4c, Supporting Information). The peaks at 228.6 and 231.8 eV were attributed to the Mo^4+^ 3d_5/2_ and Mo^4+^ 3d_3/2_ of metallic 1T‐MoS_2_, respectively (Figure S4d, Supporting Information). Additionally, the iron doping concentration in the MF nanoflowers, as measured by inductively coupled plasma mass spectrometry, was 8.34 wt.%.

To confer bacteria‐targeting capability and enhance physiological stability, MF nanoflowers were modified with MBA and HA through the interaction between the sulfhydryl group and MF. The Fourier transform infrared (FTIR) spectrum of MF‐MBA revealed the disappearance of the characteristic peak of MBA at 2565 cm^−1^, which corresponds to the S‐H vibration, indicating the successful modification of MBA onto MF nanoflowers (Figure 1b).^[^ ^15^ ^]^ Moreover, the appearance of the Ultraviolet–visible spectroscopy (UV–vis) absorption peak of the benzene ring at 255 nm further confirmed the FTIR results (Figure S5, Supporting Information). To further modify the surface of MF‐MBA with HA, SH‐HA was subsequently synthesized through an amide reaction between the amino group of cysteamine and the carboxyl group of HA. The signal at 2.82 ppm corresponding to ‐CH_2_ was detected in SH‐HA but not in HA, confirming the successful preparation of SH‐HA (Figure S6, Supporting Information). Furthermore, the increasing hydrodynamic size indicated the successful construction of MFBH (Figure 1c). The physiological stability of MFBH was also enhanced, which corroborated the successful modification of HA (Figure 1d). The stability of MFBH was further proven by the constant particle size (Figure 1f).

### 2.2 Functional Characterization of MFBH

The peroxidase (POD)‐like catalytic activity of MFBH was investigated with 3,3′,5,5′‐tetramethylbenzidine (TMB) colorimetric assay in sodium acetate buffer (pH = 4.5). The principle of this assay is that MFBH can oxidize colorless TMB to generate blue oxidized TMB in the presence of hydrogen peroxide (H_2_O_2_) owing to the generation of ·OH.^[^ ^16^ ^]^ The strength of the POD‐like catalytic activity was reflected by UV‐vis absorption at 652 nm. As shown in Figure S7 (Supporting Information), obvious peaks at 652 nm were observed in the MoS_2_/Fe@HA (MFH) and MFBH groups, whereas no peak was present in the control groups, illustrating the POD‐like catalytic activity of MFH and MFBH. Furthermore, the POD‐like catalytic activity of MFBH was found to be concentration‐dependent, increasing with increasing concentrations of MFBH (0, 7.5, 15, 30, and 45 µg mL^−1^) (Figure S8, Supporting Information).

GSH is a key component of the antioxidant defense system, and it is overexpressed in sites of bacterial infection and can protect bacteria from extraneous oxidative stress by scavenging ROS.^[^ ^17^ ^]^ Therefore, breaking down the antioxidant defense system by consuming GSH is beneficial for triggering bacterial ferroptosis‐like death. According to previous reports, MoS_2_ can promote GSH oxidation.^[^ ^18^ ^]^ Thus, the loss of GSH was explored via Ellman's assay, and the content of GSH was quantified based on UV–vis absorption at 410 nm. MFBH accelerated GSH loss due to the presence of the MoS_2_ component (Figure S9, Supporting Information). Moreover, the temperature‐dependent and concentration‐dependent GSH oxidation was further explored at room temperature. As shown in Figure S10a (Supporting Information), the GSH content in the system gradually decreased with increasing of MFBH concentration. Additionally, Figure S10b (Supporting Information) revealed that GSH consumption increased gradually over time, reaching ≈80% after 4 h. These results indicated the remarkable GSH consuming activity of MFBH, which guaranteed the amplification of oxidative‐stress‐induced ferroptosis‐like death.

### 2.3 Characterization of Biocompatibility In Vitro and In Vivo

Excellent biosafety is crucial for the biological application of nanodrugs. Therefore, a comprehensive evaluation of the biocompatibility of MFBH was carried out by cytotoxicity assay, hemolysis assay and histological analysis. The cytotoxicity of MFBH was assessed via the methylthiazolyldiphenyl‐tetrazolium bromide assay. As shown in Figure 1e, cell viability did not significantly decrease and was maintained at a high level (≈80%) after treatment with various concentrations of MFBH (0–30 µg mL^−1^), indicating that MFBH exhibited low cytotoxicity. Hemocompatibility was evaluated using a 2% suspension of mouse‐derived erythrocytes with pure water and saline as positive and negative controls, respectively. As shown in Figure 1g, the hemolysis ratios were less than 5% for all the investigated concentrations of MFBH, demonstrating high hemocompatibility.

Given that intravitreal injection is the primary route for treating endophthalmitis, we injected MFBH into the posterior segments of normal eyes to evaluate its biocompatibility in vivo. As shown in Figure 1h, there was no significant difference between the MFBH groups and the control group. After treatment with compound tropicamide eye drops, all the pupils were dilated and no signs of pupillary adhesion were observed. Then, hematoxylin and eosin (H&E) staining was employed to further analyze the effect of MFBH on ocular tissues (Figure 1i,j). As expected, no obvious inflammation was observed in the MFBH groups and the ocular tissues including the conjunctiva, ciliary body and retina, were intact. These results were consistent with those of the control group, indicating that MFBH did not affect the eyeball. Taken together, these results demonstrated that MFBH possesses excellent biocompatibility and has potential to be used as a nanodrug for the clinical treatment of endophthalmitis.

### 2.4 MFBH with Peptidoglycan‐Targeted Antibacterial Activity, including Four Samples of Clinical Isolates In Vitro

**Figure 2:**
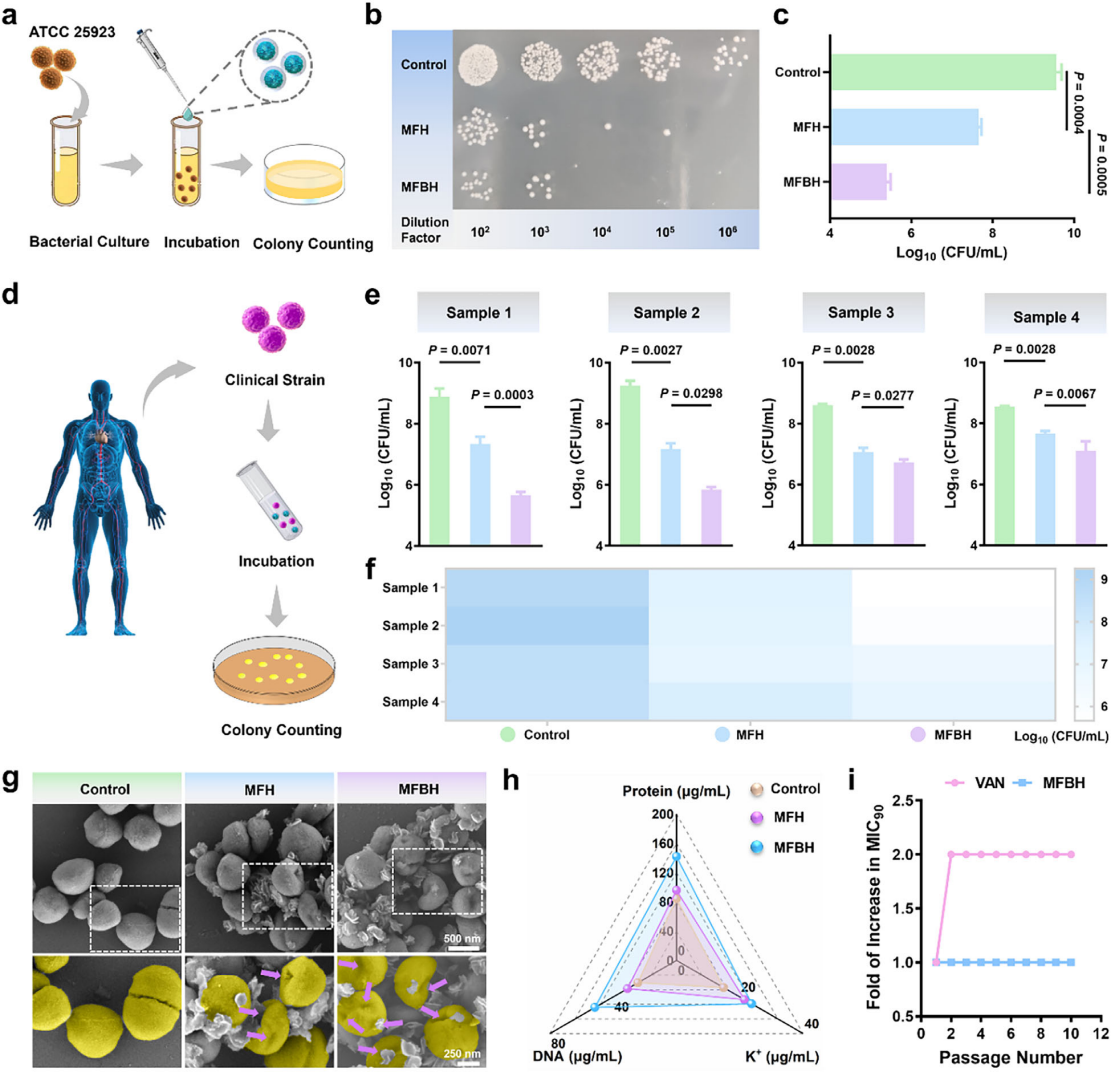
The peptidoglycan‐targeted catalytic antibacterial capacity of MFBH in vitro. a) Schematic diagram of the process of detecting the antibacterial ability of MFBH against *S.aureus* ATCC 25 923. b) Representative photographs of *S.aureus* ATCC 25 923 treated with different artificial biocatalysts. c) The survival rates of *S.aureus* ATCC 25 923 treated with different artificial biocatalysts. d) Schematic diagram of the process of detecting the antibacterial ability of MFBH against clinically isolated *S. aureus*. The survival rates of clinically isolated *S. aureus* treated with different artificial biocatalysts are presented in e) histograms and f) heatmaps. g) SEM images of *S.aureus* ATCC 25 923 after treatment with different artificial biocatalysts. The purple arrows indicate folds or breaks in the bacterial surface. h) Comprehensive evaluation of the exudation of intracellular components, including potassium ion (K^+^), protein and DNA. i) Folds of increase in MIC_90_ of MFBH through serial passages of growth inhibition assays against *S.aureus* ATCC 25 923. (Data are presented as mean ± SD. Statistical significance was assessed using Student's *t*‐tests. *n* = 3.).

The antibacterial activity of MFBH against ATCC 25 923 standard strain and clinically isolated strains of *S. aureus* was further investigated. MFH presented an antibacterial effect against *S. aureus* ATCC 25 923, probably due to the inherent antibacterial activity of the MF nanoflowers. Comparatively, the number of bacterial colonies in the MFBH groups was significantly reduced, which can be attributed to the ability of MBA to target peptidoglycan on the bacterial surface (**Figure** **2**a,b). As further supported by the corresponding histograms, only a 1.9 log_10_ CFU mL^−1^ reduction was observed in the MFH groups, whereas the MFBH groups showed a reduction exceeding 3 log_10_ CFU mL^−1^ (Figure 2c). These results suggested the excellent antibacterial activity of MFBH. Additionally, the antibacterial activity of MFBH was positively correlated with both incubation time and concentration (Figure S11, Supporting Information). Also, we evaluated the killing effects of MFBH on clinically isolated *S. aureus* named samples 1, 2, 3 and 4, respectively. Surprisingly, MFBH also exhibited superior antibacterial effects against these clinical strains, as evidenced by significantly reduction in bacterial colonies (Figure 2d–f; Figure S12, Supporting Information). These findings highlight the potential of MFBH for clinical application in the treatment of *S. aureus* infections.

Afterward, we examined the antibacterial effect of MFBH at the cellular level. First, the micromorphological changes in *S. aureus* after treatment were revealed by scanning electron microscopy (SEM). As shown in Figure 2g, an intact and smooth spherical morphology was observed in the control groups. However, after MFH treatment, the bacterial surface appeared deformed. Notably, more bacteria with wrinkled surfaces were seen in the MFBH groups, suggesting a more potent cell‐destroying effect. Additionally, after treatment, a greater number of MFBH than MFH were found to be adhered to the bacterial surface, suggesting that MFBH had a peptidoglycan‐targeted effect, which was consistent with the findings of our previous study.^[^ ^15^, ^19^ ^]^ Additionally, considering that the peptidoglycan‐targeted effect of MFBH is derived from MBA, MF‐MBA was chosen to further confirm the bacterial targeting ability of MFBH using the solution‐turbidity method.^[^ ^19b^ ^]^ As shown in Figure S13 (Supporting Information), the %OD_600_ (the relative optical density at 600 nm) of the MF‐MBA groups increased rapidly after 30 min and consistently remained higher than that of both the MF groups and the control groups. This indicated that the addition of MF‐MBA facilitated the rapid sedimentation of *S. aureus*. The %OD_600_ of the MF groups was higher than that of the control groups, likely due to nonspecific adhesion resulting from the rough surface. The increase in %OD_600_ observed in the control groups may be attributed to gravitational effects.

The degree of damage to the bacterial cell membrane and wall was assessed by quantifying the leakage of intracellular components, including ions, proteins and DNA. As illustrated in Figure 2h, the treatment with MFH resulted in a more significant release of intracellular components compared to the control groups. Remarkably, the exudation of intracellular components was even higher in the MFBH groups. This implied that MFBH treatment caused more severe damage to the cell membrane, which is in agreement with the micromorphological changes observed via SEM. Besides, it is worth mentioning that prepared artificial biocatalyst not only induced efflux of intracellular components, but also exhibited damaging effects on bacterial DNA (Figure S14, Supporting Information).

Given the risk of the appearance of drug‐resistant strains induced by antibiotic misuse or overuse, we investigated the impact of MFBH on the development of bacterial resistance. The results indicated that no resistant bacteria appeared with repeated MFBH treatments, as demonstrated by a constant minimum concentration that inhibited the growth of 90% inoculated bacterial cells (MIC_90_) over 10 treatment passages. Conversely, the MIC_90_ for VAN against *S. aureus* increased 2‐fold, indicating the development of resistance (Figure 2i). In conclusion, these data suggested that MFBH not only exhibited significant peptidoglycan‐targeted bactericidal ability against *S. aureus* and related clinical strains, but also effectively inhibited the development of bacterial resistance.

### 2.5 Therapy for Bacterial Endophthalmitis

**Figure 3:**
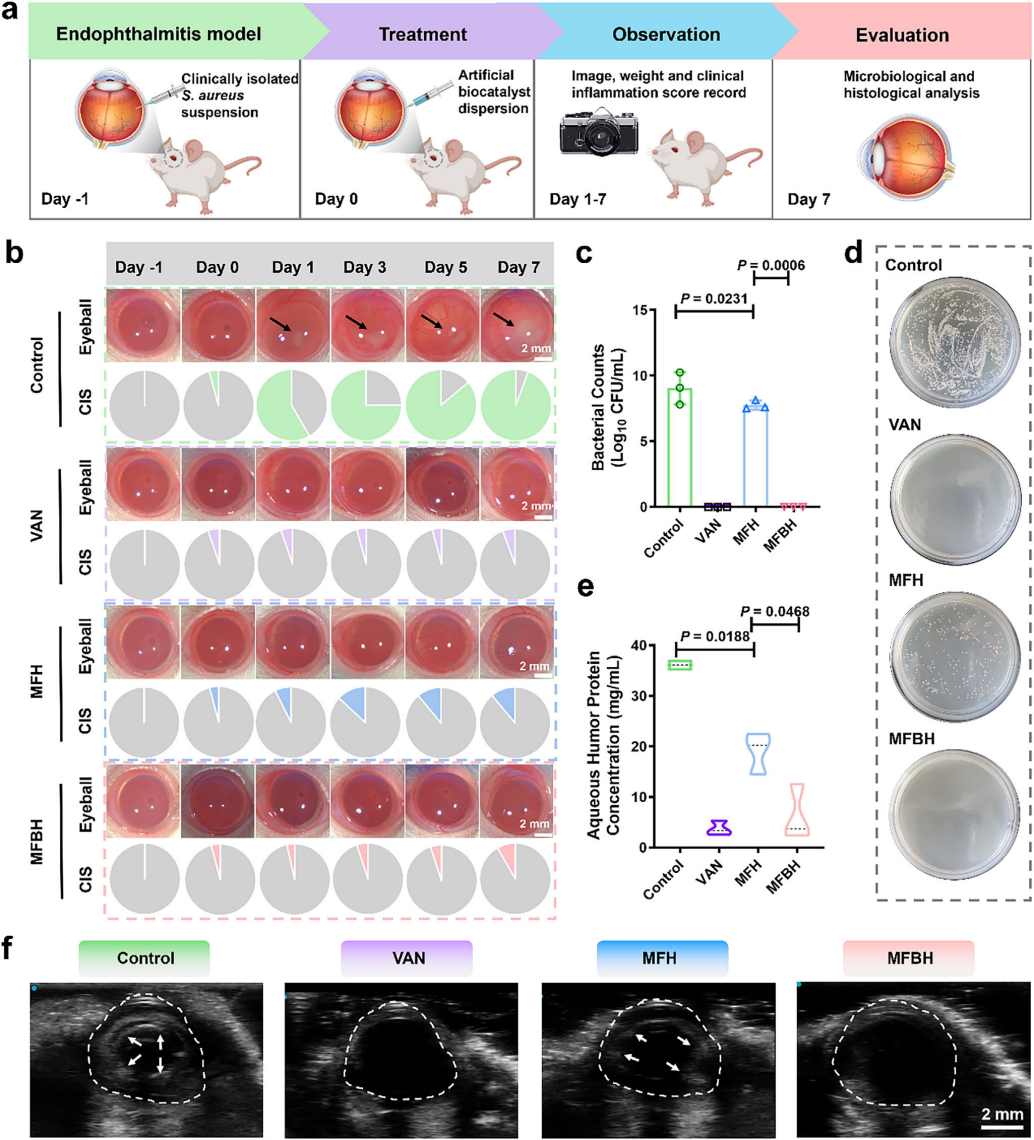
The treatment of bacterial endophthalmitis with MFBH in vivo. a) Experimental scheme for the treatment of bacterial endophthalmitis. b) Representative photographs and CIS of rat eyes after administration on days −1, 0, 1, 3, 5 and 7. The black arrows indicate fibrinous exudations. c) Bacterial colony counts and d) corresponding plate images of the vitreous humor from the rat eyes on day 7. e) The protein concentration of aqueous humor on day 7. f) B‐ultrasound images of rat eyes on day 7 post‐administration. The area circled by the white dotted line is the eyeball area. The white arrow points to the high‐density areas. (Data are presented as mean ± SD. Statistical significance was assessed using Student's *t*‐tests. *n* = 3.).

Considering its superior antibacterial effects and biocompatibility in vitro, a bacterial endophthalmitis model was established in Sprague‐Dawley (SD) rats using clinically isolated *S. aureus* (Sample 2), and this model was used to assess the therapeutic efficacy of MFBH in vivo. Intravitreal injection was chosen as the route of administration because it is the primary route for treating bacterial endophthalmitis in the clinic.^[^ ^20^ ^]^ Specifically, 100 CFU of clinically isolated *S. aureus* (Sample 2) was injected into the vitreous cavity of SD rats and incubated for 24 h. After successful model establishment, a single intravitreal injection of MFBH was administered for treatment considering the trauma associated with intraocular injections, and the total treatment period was 7 days (**Figure** **3**a). VAN, which is a clinically preferred therapeutic agent, was chosen as the positive control to assess the therapeutic effect of MFBH in treating bacterial endophthalmitis.

As shown in the real‐time ocular images and the corresponding clinical inflammation scores (CIS), obvious fibrinous exudations, conjunctival hyperemia, corneal edema and extensive purulent secretion, which are symptoms of endophthalmitis, were observed in the control groups (Figure 3b; Figure S15, Supporting Information). Over time, these symptoms worsened, as reflected by increased CIS, due to bacterial proliferation and the associated host immune response. In contrast, the MFH groups exhibited minor symptoms and lower CIS, demonstrating the antibacterial effects of MFH in vivo. Notably, there were no obvious fibrinous exudations, corneas were clearer, conjunctivas were normal and CIS was lowest after treatment with MFBH, and these effects were comparable to those of VAN. The above findings preliminarily illustrated that MFBH exhibited ultra‐efficient antibacterial capacity, which could effectively inhibit the progression of infection by rapidly killing pathogenic bacteria.

To further verify the antibacterial effects of MFBH in vivo, the rats were euthanized and the eye was enucleated on day 7. The residual bacteria in the vitreous cavity were counted by standard plate counting. The number of bacteria in each group was shown in Figure 3c,d, the number of bacterial colonies was obviously reduced in the MFH groups compared with the control groups. Accordingly, the number of residual bacterial colonies decreased by 1.3 log_10_ CFU mL^−1^. Notably, no bacterial colonies were detected after treatment with MFBH, indicating the high bactericidal capacity of MFBH in vivo. Additionally, aqueous humor protein levels were also measured to evaluate the therapeutic efficacy, as bacterial endophthalmitis‐induced inflammation can disrupt the blood aqueous barrier, leading to increased protein levels in the aqueous humor.^[^ ^5f^ ^]^ As exhibited in Figure 3e, high proteins levels in the aqueous humor were observed in the control groups, with a concentration of 36.08±0.87 mg mL^−1^. Conversely, the MFH groups presented lower protein levels, and the corresponding concentration was 19.04±4.11 mg mL^−1^. The lowest aqueous humor protein levels were observed in the MFBH groups, and these levels were similar to those in the VAN‐treated groups. These results confirmed that MFBH possessed excellent therapeutic effects on bacterial endophthalmitis, and that it prevented the occurrence of severe intraocular injuries. Furthermore, the high‐density areas in the vitreous cavity and the anterior chamber were lower in the MFBH and VAN groups than that in the other groups, further supporting the above conclusion (Figure 3f).

### 2.6 Histological Analysis

**Figure 4:**
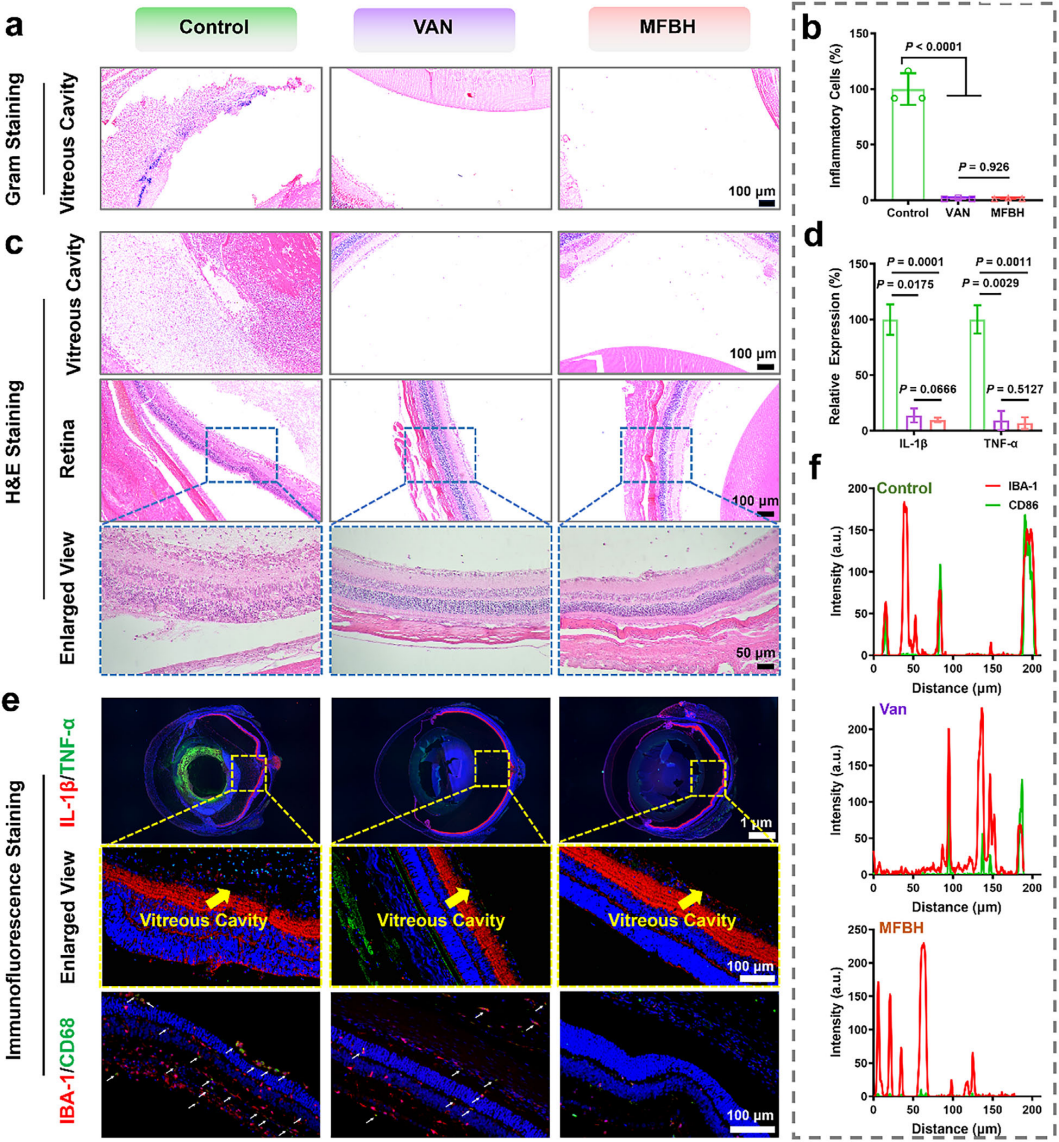
Pathological histology analysis of rat eyes on day 7. a) Gram staining of the vitreous cavity. b) Quantitative analysis of inflammatory cells in the vitreous cavity. c) H&E staining of the vitreous cavity and retina. d) Quantitative analysis of the relative expression levels of IL‐1β and TNF‐α in the vitreous cavity. e) Immunofluorescent staining of rat eyes with different treatments. The yellow arrows point to the position of vitreous cavity and the white arrows indicate the location of IBA‐1^+^/CD86^+^ in the retina. f) Fluorescence co‐localization analysis of IBA‐1 and CD86 in the retina. (Data are presented as mean ± SD. Statistical significance was assessed using Student's *t*‐tests. *n* = 3.).

Pathological histology analysis was performed to further evaluate the therapeutic effect of MFBH. As previously reported, the secretion of toxins and exposure to bacterial components, such as peptidoglycan and teichoic acids, induce strong inflammatory responses, ultimately destroying intraocular tissues.^[^ ^5^, ^21^ ^]^ Therefore, we first examined the levels of bacteria in ocular tissues via Gram staining. The results showed that MFBH effectively eliminated invading *S. aureus*, and virtually no bacteria were present in the vitreous cavity, which was consistent with previous standard plate count results (**Figure** **4**a). Then, the eye sections were examined via H&E staining to assess the inflammatory response. In the control groups, many inflammatory cells were found in the vitreous cavity (Figure 4c), conjunctiva and ciliary body (Figure S16, Supporting Information). However, almost no neutrophil infiltration was observed at the same sites after MFBH treatment. Meanwhile, the corresponding quantitative data also revealed that the degree of inflammatory cell infiltration into the vitreous cavity dramatically decreased after MFBH treatment (Figure 4b). Similarly, retinal damage, such as retinal detachment and the dissolution of retinal cells, was found only in the control groups (Figure 4c). Severe inflammation and retinal damage could be explained by the presence of large amounts of bacteria and toxins in the eye. Fortunately, MFBH treatment rapidly cleared bacteria from the eye, preventing the development of robust inflammation, and thereby protecting the retinal structure. Furthermore, the expression of proinflammatory factors, such as tumor necrosis factor‐α (TNF‐α) and interleukin‐1β (IL‐1β), in the vitreous cavity was measured by immunofluorescence staining. As shown in Figure 4d,e, almost no fluorescence was observed in the vitreous cavities of the MFBH‐treated groups compared with those of the control groups, indicating a weaker inflammatory response and reduced bacterial infection in the MFBH group. This result was consistent with the H&E staining results. Notably, the inflammatory response in the MFBH groups was similar to that in the VAN groups, suggesting that these agents exert similar therapeutic effects.

VAN has been reported to cause retinal toxicity during the treatment of endophthalmitis. To address this concern, we further evaluated the potential retinal toxicity of the prepared artificial biocatalyst by immunofluorescence staining. Microglia, which constitute up to 90% of glial cells in the retina, serve as key indicators of inflammation and retinal degradation when activated. Ionized calcium binding adaptor molecule 1 (IBA‐1) is commonly used as a marker of microglia, and the co‐localization of IBA‐1 and CD86 (IBA‐1^+^/CD86^+^) indicates microglial activation.^[^ ^22^ ^]^ As seen in the corresponding results in Figure 4e, a large number of IBA‐1^+^/CD86^+^ co‐localized signals were observed in the eyes of the control groups, likely due to extensive bacterial colonization and severe inflammatory infiltration. Meanwhile, the VAN groups also displayed distinct co‐located signals. In contrast, the IBA‐1^+^/CD86^+^ expression level was undetectable in the MFBH groups. The results were further confirmed by the corresponding fluorescence co‐localization data (Figure 4f). These findings indicate that MFBH does not cause retinal toxicity during the treatment of endophthalmitis. In addition, changes in body weight and results of H&E staining of major organs demonstrated the biosafety of MFBH (Figures S17 and S18, Supporting Information). Collectively, these evidences fully proved the remarkable therapeutic effect of MFBH in vivo. MFBH not only efficiently cleared bacteria to avoid the induction of severe inflammatory reactions, but also did not cause retinal toxicity, showing great potential for clinical translation.

### 2.7 Assessment of Long‐Term Efficacy and Safety

The therapeutic outcomes of MFBH and its potential effects on other tissues were further evaluated by extending the treatment period. First, changes in the eyes were recorded over over a period of 14 days. As shown in Figure S19 (Supporting Information), fibrous exudation (black arrow) was observed in the control groups on day 1. Along with persistent infection, the eyes presented severe corneal edema, fibrous exudation, obscure iris vessels and caligo pupillae. In contrast, the normal ocular structures, such as cornea, conjunctiva, iris and pupil, were observed in the MFBH groups. Notably, even after the treatment period was extended to 14 days, the eyes in the MFBH groups remained normal without obvious lesions. Subsequently, the rats were euthanized on day 14 and the eyes were enucleated for H&E staining. Many inflammatory cells and damaged ocular tissues were found in the control groups but not in the MFBH groups (Figure S20a, Supporting Information). These results confirmed that MFBH possessed excellent therapeutic effects in the treatment of bacterial endophthalmitis.

Furthermore, the safety of MFBH was evaluated through H&E staining of major organs and hematology analysis. H&E staining of the major organs, including the heart, liver, spleen, lung, and kidney, revealed no significant pathological abnormalities (Figure S20b, Supporting Information). Hematology analysis, including serum biochemistry and routine blood tests, revealed that all the parameters remained within normal ranges (Figure S21, Supporting Information). In addition, the weight changes of the rats within 14 days were within the acceptable range (Figure S22, Supporting Information). The ocular clearance rate of MFBH was investigated by monitoring Mo content in the MFBH‐treated eyes. Figure S23 (Supporting Information) showed that the excretion rate of MFBH was ≈70% after 24 d of drug administration. The above results demonstrated the long‐term safety of MFBH. In summary, MFBH exhibited excellent efficacy and biocompatibility, which is consistent with previous findings.

### 2.8 Antibacterial Mechanism Investigation

**Figure 5:**
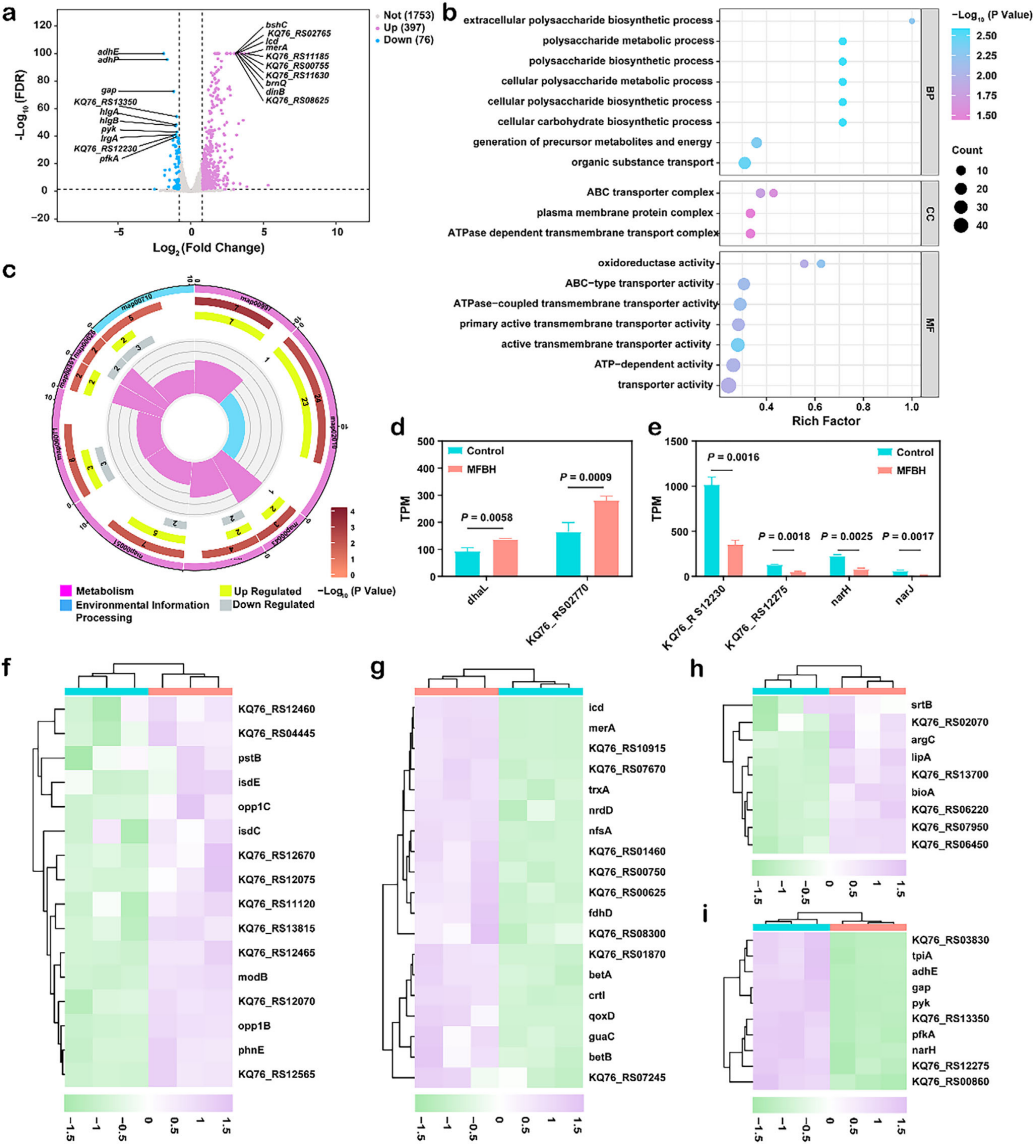
Transcriptomic changes of *S. aureus* following MFBH treatment. a) Volcano plot analysis of DEGs. b) GO analysis and c) KEGG analysis of DEGs. BP: biological process; CC: cellular component; MF: molecular function. d) DEGs associated with cell membrane and lipid peroxidation. e) DEGs associated with TCS. f) Heatmap of genes associated with iron transport. g) Heatmap of genes associated with oxidative stress. h) Heatmap of genes associated with GSH consumption. i) DEGs associated with energy metabolism. (Data are presented as mean ± SD. Statistical significance was assessed using Student's *t*‐tests. *n* = 3.).

The potential antibacterial targets and corresponding antibacterial mechanisms of the prepared artificial biocatalyst were explored through RNA‐seq analysis. A total of 2226 genes were analyzed, of which 397 genes were upregulated and 76 genes were downregulated (**Figure** **5**a; Figure S24, Supporting Information). To determine their exact functions, the differentially expressed genes (DEGs) were further analyzed using Gene Ontology (GO) enrichment analysis. This analysis revealed that the metabolic processes, adenosine triphosphate (ATP)‐binding cassette (ABC) transporter complex and oxidoreductase activity were closely associated with the mechanism by which MFBH kills bacteria (Figure 5b). Additionally, Kyoto Encyclopedia of Genes and Genomes (KEGG) enrichment analysis was also conducted to reveal that the DEGs were enriched mainly in the membrane transport, amino acid metabolism, carbohydrate metabolism, lipid metabolism and energy metabolism pathways (Figure 5c).

Next, the specific functions of these key DEGs were further analyzed to elucidate the antibacterial mechanism of MFBH. The ABC transporter system can use the energy that is generated by ATP hydrolysis to increase the bacterial uptake of iron ions, leading to intracellular iron aggregation.^[^ ^5^, ^23^ ^]^ The heatmap showed that almost all genes associated with ABC transporters, such as modB (KQ76_RS11635), opp1B (KQ76_RS12680), opp1C (KQ76_RS12675) and KQ76_RS11120, were significantly upregulated (Figure 5f). In addition, genes that are related to iron coordination entity transport, including isdE (KQ76_RS05320) and isdC (KQ76_RS05310), were also appreciably upregulated. These results indicated an increase in iron uptake by bacteria after MFBH treatment. Intracellular Fe overload can induce ROS production via Fenton chemistry, further triggering oxidative stress.^[^ ^8^, ^24^ ^]^ As shown in Figure 5g, genes that are related to oxidative stress were expressed at significantly higher levels in the MFBH groups than in the control groups.

GSH is the main antioxidant that protects bacteria from oxidative stress damage.^[^ ^25^ ^]^ Cysteine is the limiting substrate for GSH synthesis and can be produced from methionine via the transsulfuration pathway.^[^ ^26^ ^]^ Cysteine and mevalonate metabolism are closely related to the depletion of GSH. As presented in Figure 5h, genes that are related to cysteine and methionine metabolism were obviously upregulated, indicating GSH depletion. In addition, the upregulation of the glutathione peroxidase gene (KQ76_RS06220) further confirmed this conclusion. Depletion of GSH further increases the intracellular ROS levels, ultimately causing lipid peroxidation. Additionally, glycerolipids and fatty acids respond to external stress by altering cell membrane permeability and fluidity as well as lipid peroxidation.^[^ ^27^ ^]^ The DEGs of dhaL (KQ76_RS03160) and KQ76_RS02770 were significantly upregulated, indicating that MFBH improved bacterial lipid peroxidation and membrane permeability (Figure 5d).

Energy metabolism is important in biological processes and molecular functions. Figure 5i shown that energy metabolism, including pyruvate metabolism, glycolysis and nitrogen metabolism, was remarkably inhibited. Act as a critical sensory pathway, the two‐component signal transduction system (TCS) is beneficial for bacterial adaptation to extracellular environments and stresses and for increasing survival.^[^ ^8^, ^28^ ^]^ Genes related to the TCS, including KQ76_RS12230, KQ76_RS12275, narH (KQ76_RS12270) and narJ (KQ76_RS12265), were downregulated in the MFBH‐treated groups (Figure 5e). The above results indicated that MFBH disrupted energy metabolism and the TCS, thus inhibiting bacterial growth. Taken together, MFBH was able to disrupt the bacterial cell membrane, increase the intracellular iron content and interfere with redox homeostasis and energy metabolism, which was similar to the mechanism of ferroptosis death in eukaryotic cells. Thus, these results suggested that MFBH induced the bacterial ferroptosis‐like death.

**Figure 6:**
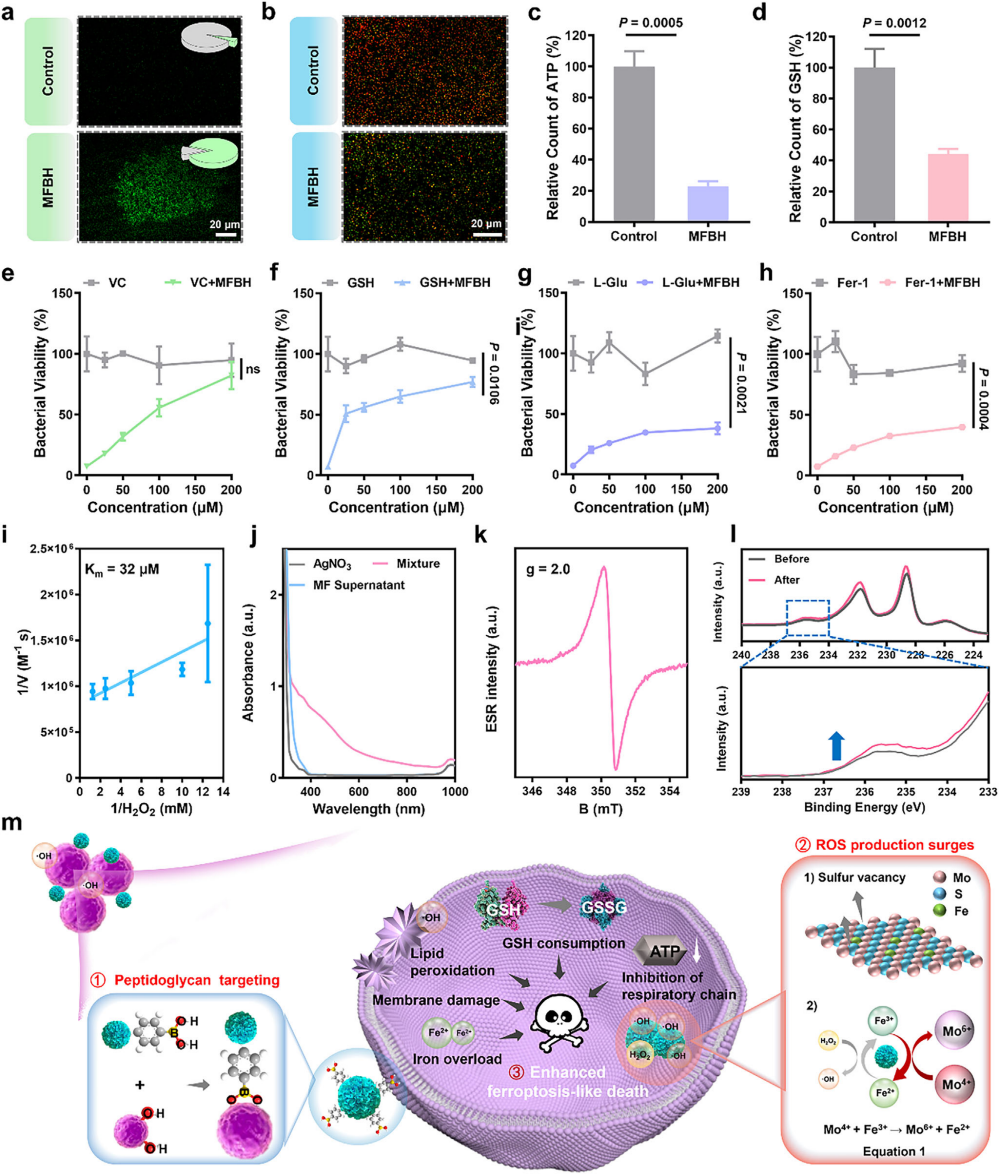
Mechanism investigation of the prepared artificial biocatalysts. CLSM images of bacteria stained with a) DCFH‐DA and b) C11‐BODIPY^581/591^. The green portion of the pie charts represent the percentage of green fluorescent area in a). c) ATP levels and d) GSH levels in bacteria with different treatments. e–h) VC, GSH, L‐Glu and Fer‐1 inhibit of MFBH‐induced bacterial death. i) Double‐reciprocal Lineweaver‐Burk plotting of the MF nanoflowers based on (Figure S27, Supporting Information). j) UV–vis spectrum of different samples. k) ESR spectrum of MF nanoflower. l) XPS spectrum of Mo 3d before and after H_2_O_2_ treatment. m) Schematic illustration of the antibacterial mechanism of MFBH. (Data are presented as mean ± SD. Statistical significance was assessed using Student's *t*‐tests. *n* = 3.).

The RNA‐seq results were further validated by investigating hallmarks of ferroptosis‐like death, including membrane damage, lipid peroxidation, respiratory chain inhibition and GSH consumption.^[^ ^9b^ ^]^ As described previously, membrane damage had been verified by SEM and the exudation of intracellular components. From **Figure** **6**a, a higher ROS level was observed after MFBH treatment. Accordingly, the MFBH groups exhibited a strong green fluorescent signal characterizing the oxidation of C11‐BODIPY^581/591^, indicating the occurrence of lipid peroxidation (Figure 6b).^[^ ^29^ ^]^ ATP, which is produced primarily by oxidative respiration, is necessary for bacteria to carry out their biological activities.^[^ ^30^ ^]^ Figure 6c displayed a sharp decrease in ATP content when MFBH was applied, suggesting the respiratory chain inhibition. Besides, significantly decreases in GSH levels were also found in the MFBH groups (Figure 6d). These results confirmed the occurrence of bacterial ferroptosis‐like death. Then, various types of ferroptosis inhibitors were added to the *S. aureus* suspensions containing MFBH to further validate the bacterial ferroptosis‐like death.^[^ ^8^, ^31^ ^]^ GSH and vitamin C (VC), which are antioxidants, inhibited the bactericidal effect of MFBH (Figure 6e,f). In addition, L‐glutamate (L‐Glu) also affect the antibacterial effects of MFBH by chelating Fe^2+^ (Figure 6g). Importantly, ferrostatin‐1 (Fer‐1) also weakened the bacterial killing effect of MFBH to some extent (Figure 6h). Images of the corresponding plates are shown in Figures S25 and S26 (Supporting Information). Based on these experiments, we conclude that MFBH effectively induced bacterial ferroptosis‐like death. Confusingly, it has been reported that 18 µM Fe^2+^ can increase bacterial lipid peroxidation and inhibit the bacterial respiratory chain, thereby triggering the bacterial ferroptosis‐like death.^[^ ^8b,c^ ^]^ In this work, the iron concentration in MFBH was 12 µM, which was lower than the aforementioned concentration. Thus, we speculate that additional factors, apart from Fe^2+^, provoked bacterial ferroptosis‐like death.

Bacterial ferroptosis‐like death is closely associated with the ROS derived from intracellular Fe^2+^ via the Fenton chemistry, primarily involving ·OH that mediate lipid peroxidation.^[^ ^9a^ ^]^ The more ·OH is produced, the more severe the degree of peroxidation. Therefore, we speculate that MFBH can generate large amounts of ·OH to trigger bacterial ferroptosis‐like death. As confirmed in Figure S7 (Supporting Information), ·OH originated from H_2_O_2_ decomposition, which was catalyzed by MFBH with POD‐like catalytic activity. The strong H_2_O_2_ affinity contributes to ·OH production.^[^ ^32^ ^]^ As depicted in Figure 6i and Figure S27 (Supporting Information), the Michaelis‐Menten constant (K_m_) value of MF for H_2_O_2_ was 32 µM, which was lower than the K_m_ values reported for most other nanozymes (Table S1, Supporting Information). These results proved the high affinity of MF for H_2_O_2_. Subsequently, we further explored the reason for this conclusion. It has been that unsaturated S atoms on the MoS_2_ surface can be removed by binding with protons to form hydrogen sulfide (H_2_S), resulting in the formation of sulfur vacancies.^[^ ^10a^ ^]^ These vacancy defects favor the adsorption of H_2_O_2_, which in turn enhances the H_2_O_2_ affinity.^[^ ^10^, ^33^ ^]^ Following this, the sulfur vacancies on the surface of the MF were detected via an H_2_S generation assay and electron spin resonance (ESR). H_2_S was identified with AgNO_3_ because Ag^+^ reacts with H_2_S to form a silver sulfide (Ag_2_S) precipitate.^[^ ^34^ ^]^ In Figure 6j, only the mixture group showed a wide range of UV‐visible absorption curves caused by Ag_2_S, proving the presence of H_2_S. The gradual decrease in the amount of precipitate with increasing number of washes further indicated the formation of sulfur vacancies (Figure S28, Supporting Information). Additionally, the signal at g = 2.0 corresponding to a sulfur vacancy, was also observed in the ESR spectra of MF (Figure 6k). Figure S29 (Supporting Information) revealed that MF catalyzed the generation of ·OH.

In addition to the affinity, the conversion of Fe^3+^ to Fe^2+^ also limits the process of H_2_O_2_ decomposition. Fortunately, Mo^4+^ has been reported to facilitate this conversion.^[^ ^35^ ^]^ More interestingly, the formation of sulfur vacancies is conducive to the exposure and activation of Mo^4+^.^[^ ^10a^ ^]^ The oxidation‐reduction reaction is shown in **Equation 1** and was confirmed by XPS. The elemental valence states of MF after the reaction were analyzed by XPS (Figure S30, Supporting Information). In Figure 6l, the Mo 3d XPS spectrum after the reaction exhibited a stronger peak at 236 eV, which corresponds to Mo^6+^, proving the oxidation of Mo^4+^ to Mo^6+^. The presence of Mo^6+^ before the reaction was likely caused by the slight oxidation of the MF due to exposure to air. The stretching mode of Mo_2_‐O at 825 cm^−1^ in the Raman spectrum further indicated the reductive property of the exposed Mo^4+^ sites, which promoted the reduction of Fe^3+^ to Fe^2+^. (Figure S31, Supporting Information). In summary, the above results suggested that the generation of large amounts of ·OH was attributed to the multiple sulfur vacancies and exposed reactive Mo^4+^ on the surface of the prepared artificial biocatalyst. These factors could increase the affinity for H_2_O_2_ on the surface of the artificial enzyme and promote the reduction of Fe^3+^ to Fe^2+^. Excessive ·OH leads to oxidative damage of cell membrane, ultimately initiating the bacterial ferroptosis‐like death at low Fe^2+^ concentrations.^[^ ^30^ ^]^

Combined with the peptidoglycan‐targeted ability confirmed by the SEM results, the specific antibacterial mechanism is illustrated in Figure 6m. First, MFBH targeted pathogens by establishing borate bonds between MBA and peptidoglycan on the bacterial surface. Subsequently, it catalyzed the decomposition of H_2_O_2_ to generate ·OH through its inherent POD‐like catalytic activity. Notably, the presence of sulfur vacancies and exposed reactive Mo^4+^ on the surface of the prepared artificial biocatalyst greatly promoted the H_2_O_2_ decomposition to generate a large amount of ·OH by improving the H_2_O_2_ affinity and facilitating Fe^3+^ reduction. The excess ·OH further led to oxidative damage to the cell membrane, inducing bacterial ferroptosis‐like death, ultimately resulting in bacterial death.

## 3 Conclusion

In summary, a bacteria‐specific artificial biocatalyst, namely MFBH, which is composed of MBA/HA‐modified MF nanoflower, was developed for treating bacterial endophthalmitis. MFBH exhibited high‐efficiency peptidoglycan‐targeted catalytic antibacterial capacity against ATCC 25 923 standard strain and clinically isolated strains of *S. aureus*, thereby protecting intraocular tissues from severe inflammation and bacterial invasion. Importantly, the therapeutic efficacy of MFBH, which was achieved through a single intravitreal injection, was similar to that of VAN in vivo. Distinguished from antibiotic, the antibacterial mechanism of MFBH was as follows: First, MFBH targeted peptidoglycan on the pathogenic bacterial surface via MBA and catalyzed the decomposition of H_2_O_2_ to generate ·OH through its inherent POD‐like catalytic activity. The presence of sulfur vacancies and exposed reactive Mo^4+^ on the surface of the prepared artificial biocatalyst promoted the ·OH production by improving the affinity for H_2_O_2_ and facilitating the reduction of Fe^3+^, respectively. Subsequently, excess ·OH caused oxidative damage to the bacterial cell membrane, triggering a robust ferroptosis‐like death process at low doses and ultimately leading to bacterial death. Notably, in contrast to conventional antibiotics, MFBH inhibited the development of bacterial resistance and caused no retinal toxicity after intravitreal injection. Overall, the exceptional peptidoglycan‐targeted catalytic bactericidal performance of MFBH demonstrated its potential for clinical translation.

## 4 Experimental Section

### Ethical Approval

The experiments were performed in accordance with the Guidelines for Care and Use of Laboratory Animals of China Pharmaceutical University. All animal experiments were approved by the Animal Ethics Committee of School of Pharmacy, China Pharmaceutical University.

### Statistical Analysis

*Pre‐processing of data*: In the cytotoxicity experiment, the cell viability value of the control groups was recorded as 100%, and the cell viability of the experimental groups were calculated on this basis. In the hemolysis experiment, the average absorbance of blood in ultrapure water and saline was used as the hemolysis ratio of 100% and 0%, respectively. In histological analysis, the extent of inflammation and the relative expression levels of IL‐1β and TNF‐α of the control groups were recorded as 100%, and the corresponding values for the experimental groups were calculated based on this reference. Image J was used for image signal processing and quantitative statistics. For mechanism investigation, the ATP levels and GSH levels of the control group was noted as 100%, and the corresponding values for the MFBH groups were calculated based on this value as the standard.

### Data Presentation

All experimental data were statistically analyzed, and the results were presented as mean ± standard deviation (SD). Sample size (n) for each statistical analysis. The sample size for each statistical analysis was 3. Statistical methods used to assess significant differences. It was used two‐sided Student's *t*‐tests to analyze the statistical difference in the comparisons between two groups. One‐way analysis of variance (ANOVA) with post‐hoc Tukey's test was used to analyze the statistical difference between two or more groups. In all cases, a P value less than 0.05 was considered to be statistically significant. Software used for statistical analysis. GraphPad Prism 8 software (GraphPad Software Inc.).

## Conflict of Interest

The authors declare no conflict of interest.

## Supporting information



Supporting Information

## Data Availability

The data that support the findings of this study are available from the corresponding author upon reasonable request.
