# Supplementary material for: A Tailored Artificial Biocatalyst for Bacterial Endophthalmitis Therapy via Enhanced Ferroptosis‐Like Death
Source: Adv Sci (Weinh). 2025 Jun 10;12(33):e04601. doi: 10.1002/advs.202504601 (PMC12412499; doi:10.1002/advs.202504601)
Supplement: Supplementary file 1 — Supporting Information [file ADVS-12-e04601-s001.docx]

Supporting Information

A Tailored Artificial Biocatalyst for Bacterial Endophthalmitis Therapy via Enhanced Ferroptosis-Like Death

Caixia Sun, Yingying Jiang, Shibo Zhang, Zhipeng Ding, Lijun Pu, Xinmei Liu, Jun Yang, Xiangkai Zhuge, Jianjun Dai,* and Yanmin Ju*

**Experimental Section**

*Chemicals*: Ammonium molybdate tetrahydrate ((NH_4_)_6_Mo_7_O_24_·4H_2_O), thiourea (CH_4_N_2_S), iron trichloride hexahydrate (FeCl_3_·6H_2_O), cysteamine (C_2_H_7_NS), vitamin C (VC), L-glutamate (L-Glu), hyaluronic acid (HA), EDC·HCl and N-Hydroxy succinimide (C_4_H_5_NO_3_) were purchased from Shanghai Macklin Biochemical Technology Co., Ltd. (Shanghai, China). Mercaptophenylboronic acid (MBA) were supplied from Titan (Shanghai, China). Glutathione (GSH) was purchased from Beyotime Biotechnology Co., Ltd. (Shanghai, China). Ferrostatin-1 (Fer-1) was provided by Shanghai yuanye Bio-Technology Co., Ltd. (Shanghai, China).

*Preparation of MoS_2_/Fe (MF) Nanoflowers, MoS_2_/Fe@HA (MFH) and MoS_2_/Fe@MBA@HA (MFBH**)*: MF nanoflowers were synthesized by hydrothermal method. Typically, (NH_4_)_6_Mo_7_O_24_·4H_2_O (353 mg) and CH_4_N_2_S (651 mg) were dispersed in deionized water (5 mL) and stirred for 30 min. Then, the 5 mL FeCl_3_·6H_2_O aqueous solution (13 mм) was slowly dropped into the previous solution. The color of the mixed solution changed from blue to light yellow. After 30 min, the pH value of mixed solution was adjusted to 2 by adding hydrochloric acid solution. Next, the mixed solution was then transferred into a Teflon-lined autoclave chamber and heated at 200 ℃ for 10 h. Finally, the MF nanoflowers were collected by centrifugation and washed with deionized water.

MFH and MFBH were synthesized by following procedures with surface modification based on MF nanoflowers. First, SH-HA was prepared by an amide reaction between C_2_H_7_NS and hyaluronic acid. In a typical synthesis, hyaluronic acid (50 mg), EDC·HCl (48 mg), C_4_H_5_NO_3_ (17.25 mg) and 5 mL of deionized water were placed into a 100 mL ﬂask with vigorous mixing. After 2 h, C_2_H_7_NS (14.5 mg) was added, and the mixture was allowed to react for an additional 4 h. Then, the mixed solution was puriﬁed by dialysis to remove unreacted reagents. To obtain MFBH, a mixture of C_6_H_7_BO_2_S (200 μL, 65 mм) and MF nanoflower solution (1 mL, 1 mg mL^-1^) was continuously stirred at room temperature for 12 h, followed by the addition of above prepared SH-HA solution (286 μL) for reacting for another 24 h. Similarly, MFH was synthesized by adding the above prepared SH-HA solution (286 μL) to MF nanoflowers solution (1 mL, 1mg mL^-1^) and reacting for 24 h at room temperature.

*Characteristics*: The morphologies of materials were observed by transmission electron microscopy (FEI Tencnai T20). The crystal structure was revealed by powder X-ray diffraction (D/max-2400) (XRD) with a 1.54 Å Cu Kα source. The chemical states and valence bands of MF nanoflower were measured by X-ray photoelectron spectroscopy (XPS) using PerkinElmer PHI 5600. Dynamic light scattering of MF nanoflower MF nanoflower were recorded by Omni multiangle particle size. The type of functional groups on the surface of MF nanoflower were unveiled by Fourier transform infrared using OPUS5.5 software for 4000-500 cm^-1^ with a resolution of 4 cm^-1^. Ultraviolet-visible (UV-vis) absorption spectra were obtained by UV-1800 (Shimadzu, Japan) and SpectraMax M3 (Molecular Devices, United States). Reman spectra were collected from DXR2xi (Thermo Scientific, United States). Electron spin resonance (ESR) were conducted on Bruker A300 spectrometer. Fluorescence images of bacteria in vitro were collected by LSM 900 (Zeiss, Germany). Rat eyeballs were recorded by DSZ2000X (Chongqing UOP Photoelectric Technology Co., China) equipped with a CCD and DNi8 (Leica, Germany).

*Assessment of Biocompatibility In Vitro and In Vivo:*

Cytotoxicity assay: Human keratinocytes cells (Hacat) were used as representatives for mammalian host cell lines to evaluate the cytotoxicity of MFBH based on Methylthiazolyldiphenyl-tetrazolium bromide (MTT) assay. Briefly, Hacat cells were planted in 96-well plates at a density of 10^4^ cells/well and cultured in Dulbecco’s modiﬁed Eagle’s medium supplemented with 10% fetal bovine serum, penicillin (100 units mL^-1^) and streptomycin (100 μg mL^-1^) for 24 h (37 °C, 5% CO_2_). Afterward, the cells were treated with various concentrations of MFBH nanoflowers dispersions for 12 h, followed by washing with sterile phosphate buffer saline. Then, 150 μL MTT solution (0.5 mg mL^−1^) was added with further incubation for 4 h at 37 °C. To dissolve the formed crystals during the MTT assay, 150 μL of dimethyl sulfoxide was added. Finally, the absorbance values of supernatant from all samples were measured at 490 nm by a microplate spectrophotometer to indicate cell viability.

Hemolysis assay: Blood from healthy C57BL/6 mice was utilized to evaluate the hemocompatibility of MFBH nanoflowers. Specifically, 50 μL of 2% red blood cell suspension was mixed with different concentrations of MFH dispersions (0.5 mL) and incubated at 37 °C for 3 h. The positive and negative control groups were pure water and saline, respectively. Finally, the absorbance of supernatant was collected at 540 nm. The hemolysis rate was calculated as follows:

$\text{Hemolysis }\left( \text{\%} \right)\text{=}{\text{(}\text{A}_{\text{S}}\text{-}\text{A}_{\text{N}}\text{)}}/{\text{(}\text{A}_{\text{P}}\text{-}\text{A}_{\text{N}}\text{)}}\text{×100\%}$ Equation (S1)

Where A_S_ represents the absorbance values of supernatant of samples, A_N_ is the absorbance values of saline and A_P_ is the absorbance values of pure water.

In vivo biosafety assay: The eyeballs of Sprague-Dawley (SD) rats were chosen to explore the safety of MFBH in vivo. 10 μL different concentrations of MFBH dispersions were injected into the vitreous cavity of the rat eyes, with saline as a blank control. After 7 days, photographs of the rat eyes were captured before and after compound tropicamide eye drops treatment to initially judge the state of the eyes. Subsequently, the eyeballs were collected and fixed with 4% paraformaldehyde solution for histopathology.

*Peroxidase-Like (POD-Like) Activity Assessment*: 3,3’,5,5’-tetramethylbenzidine (TMB) was employed as a chromogenic substrate to detect the POD-like activity of nanoflowers. Briefly, MFBH or MFH were added to a sodium acetate buffer (0.5 mL, pH = 4.5) containing TMB (25 μL, 40 mм), hydrogen peroxide (H_2_O_2_) (20 μL, 10 mм). The mixture was allowed to react for 2 min at room temperature. The absorbance at 652 nm was measured by UV-vis spectroscopy.

*GSH Depletion*: Ellman’s assay was taken to detect the GSH-depleting property of nanoflowers. In brief, equal volumes of nanoflowers bicarbonate buffer dispersions (5 μg mL^-1^) and GSH bicarbonate buffer solution (20 μм) were mixed and incubated at room temperature in a shaking condition (150 rpm). Then, 1 mL of supernatant was extracted and the content of GSH was determined using a GSH assay kit (Jiangsu KeyGEN Bio TECH Co., Ltd, Cat#KGT006). The time-dependent and concentration-dependent GSH depletion were investigated by varying the incubation time and MFBH concentration.

*Antibacterial Assessment In Vitro*: *Staphylococcus aureus* (*S. aureus*) (ATCC 25923) and clinically isolated *S. aureus* were selected in our experiments. The logarithmic phase *S. aureus* was diluted to 10^8^ CFU mL^-1^ (OD600 = 0.1) with saline, and then MFBH and MFH dispersions were added respectively and incubated for 12 h at 37 ℃. Finally, the bacteria mixtures were diluted and cultured on Luria-Broth medium agar for 24 h at 37 ℃. The final concentration of MFBH and MFH dispersions was 8 μg mL^-1^. The volume of culture medium was 0.5 mL. The bactericidal efficacy of MFBH against clinically isolated *S. aureus* was assessed following a similar procedure. Both the time-dependent and concentration-dependent antibacterial activities of MFBH were evaluated by varying the incubation time and concentration.

*Peptidoglycan-targeted effect of MFBH*: Equal masses of MF-MBH and MF were added to the *S. aureus* suspension and incubated at 37°C. The OD_600_ values of the supernatant were detected at predetermined time. The %OD_600_ (the relative optical density at 600 nm) was calculated according to the following equation:

$\text{\%}\text{OD}_{\text{600}}\text{=(}\text{OD}_{\text{0}}\text{-OD)÷}\text{OD}_{\text{0}}\text{×100\%}$ Equation (S2)

where OD_0_ is the initial OD_600_ of *S. aureus* suspension, and OD is the OD_600_ of *S. aureus* after being treated with MF-MBA at predetermined time.

*Mechanism Investigation:*

Transcriptome analysis: *S. aureus* was divided into two groups and treated with or without MFBH, respectively. Then, bacterial precipitation was collected according to the instructions of Personal Technology Co., Ltd. (Shanghai, China). The total RNA of *S. aureus* was extracted using the Trizol Reagent (Invitrogen Life Technologies). Quality and integrity were determined using a NanoDrop spectrophotometer (Thermo Scientific) and a Bioanalyzer 2100 system (Agilent). After removing rRNA (Zymo-Seq RiboFree Total RNA Library Kit), mRNA was treated with fragmentation buffer to obtain short fragment. Next, RNA sequencing library was constructed and sequenced via an NovaSeq 6000 platform. Finally, differentially expressed genes (|log_2_ Fold Change| > 1 and P-value < 0.05) were detected and further analyzed using Gene Ontology and Kyoto Encyclopedia of Genes and Genomes analysis, respectively. All analyses were carried out by Personal Technology Co., Ltd.

Bacterial ferroptosis-like death: MFBH dispersion was incubated with the logarithmic phase *S. aureus* at 37 ℃ for 12 h. The level of reactive oxygen species was measured using the fluorescent probe (2′,7′-Dichlorodihydroffuorescein diacetate), with detection carried out LSM 900 (Zeiss, Germany). The membrane damage was determined by scanning electron microscope and the contents outflow including potassium ion (K^+^), protein and DNA. The levels of GSH and adenosine triphosphate (ATP) were detected by a GSH assay kit (Jiangsu KeyGEN Bio TECH Co., Ltd, Cat#KGT006) and an ATP assay kit (Promega Biotechnology, Cat#TB337). The antibacterial mechanism was further explored by adding inhibitors including GSH, VC, L-Glu and Fer-1.

Kinetic assay: MF nanoflowers (10 μL, 1 mg mL^-1^) and TMB (25 μL, 40 mм) were added to the sodium acetate (NaAc) buffer (0.5 mL, pH = 4.5) containing various H_2_O_2_ concentrations. The reactions were monitored by collecting the absorbance at 652 nm in kinetic mode of spectrophotometer. The kinetic parameters including Michaelis-Menten constant (K_m_) and maximum velocity were calculated based on the Michaelis-Menten equation.

ESR measurement: MF nanoflowers (10 μL, 1 mg mL^-1^), H_2_O_2_ (20 μL, 10 mм) and 5,5-Dimethyl-1-pyrroline-N-oxide were added to the NaAc buffer (pH = 4.5) in order. The reaction volume and time were 0.5 mL and 2 min, respectively.

Generation of hydrogen sulfide (H_2_S): The washing solution of the MF nanoflowers was collected, and 200 μL of supernatant was extracted and injected into silver nitrate solution (50 μL, 3 mм). The absorbance curve of mixed solution was immediately measured using UV-vis spectroscopy for analysis the formation of H_2_S.

Mo^4+^ oxidation: MF nanoflowers (20 mL, 1 mg mL^-1^) and H_2_O_2_ (1.6 mL, 10 mм) were added to the 20 mL of NaAc buffer (pH = 4.5). After incubation for 5 min, the above solution was washed with deionized water several times. The valance changes of Mo element and the Mo^4+^ oxidation in the reaction precipitate were determined by XPS and Reman.

*Animal Experiments:*

Construction of a rat endophthalmitis model: All animal experiments were following the protocol approved by the Animal Ethics Committee of School of Pharmacy, China Pharmaceutical University. SD rats (200-250 g, male) were purchased from Nanjing Qinglongshan Animal Farm and treated in accordance with the Guidelines for Care and Use of Laboratory Animals of China Pharmaceutical University. All rats were anesthetized by intraperitoneal injection of 1 mL of 10 % chloral hydrate, and then the compound tropicamide and procaine hydrochloride were dripped into the eyes from each rat to achieve the mydriasis and local anesthesia. Next, clinically isolated *S. aureus* suspension (Sample 2, 100 CFU) was injected with a 29-gauge needle at 1 mm beyond the limbus of the temporal cornea. The needle was pulled out after 5-10 s. After incubation for 24 h, the rat model of bacterial endophthalmitis was successfully established.

Endophthalmitis therapy: The rat model of bacterial endophthalmitis was randomly subjected to four groups for the following study, including control, vancomycin (VAN), MFH and MFBH. Each group received an injection into the vitreous cavity consisting of either VAN solution (10 μL, 8 mg mL^-1^) or MFH or MFBH dispersion (10 μL, 100 μg mL^-1^). The rat treated with same volume of sterile saline was employed as the control group. On day -1, 0, 1, 3, 5 and 7, the state of ocular surface was recorded by stereomicroscope, and the clinical inﬂammation of anterior segment was scored according to Peyman’s classification.

Microbiological and inflammatory analysis: After seven days, the vitreous humor and aqueous humor were collected from each group. For microbiological analysis, the collected vitreous humor (5 μL) was diluted 200 times with sterile saline. The bacterial counts were calculated by standard plate counting method. For inflammatory analysis, the aqueous humor was centrifuged at 1800 rpm for 5 min, and the supernatant was diluted 40-fold with sterile saline. The anterior aqueous protein concentration was measured using a BCA assay kit (J Abbkine Scientific Co., Ltd, Cat#KTD3001).

Histopathological analysis and immunohistochemistry staining: After seven days, the rats were sacrificed and the eyeballs were enucleated, following by fixing with FAS eyeball fixative. Then, the eyeballs were embedded in paraffin and sectioned for hematoxylin and eosin (H&E) staining and immunofluorescent staining. Finally, the images of all sections were taken using DNi8 (Leica, Germany).

B-ultrasound imaging: To further evaluate the therapeutic effect, Vevo LAZR small animal ultrasound/photoacoustic imaging system (Fujifilm Group, Japan) was employed to observe the vitreous cavity. Specifically, the rats were anesthetized and their eyeballs were examined using B-ultrasound at six days after treatment.

*Statistics and Reproducibility*: Pre-processing of data. In the cytotoxicity experiment, the cell viability value of the control groups was recorded as 100%, and the cell viability of the experimental groups were calculated on this basis. In the hemolysis experiment, the average absorbance of blood in ultrapure water and saline was used as the hemolysis ratio of 100% and 0%, respectively. In histological analysis, the extent of inflammation and the relative expression levels of IL-1β and TNF-α of the control groups were recorded as 100%, and the corresponding values for the experimental groups were calculated based on this reference. Image J was used for image signal processing and quantitative statistics. For mechanism investigation, the ATP levels and GSH levels of the control group was noted as 100%, and the corresponding values for the MFBH groups were calculated based on this value as the standard.

Data presentation. All experimental data were statistically analyzed, and the results were presented as mean ± standard deviation (SD).

Sample size (n) for each statistical analysis. The sample size for each statistical analysis was 3.

Statistical methods used to assess significant differences. We used two-sided Student’s t-tests to analyze the statistical difference in the comparisons between two groups. One-way analysis of variance (ANOVA) with post-hoc Tukey’s test was used to analyze the statistical difference between two or more groups. In all cases, a P value less than 0.05 was considered to be statistically significant.

Software used for statistical analysis. GraphPad Prism 8 software (GraphPad Software Inc.).


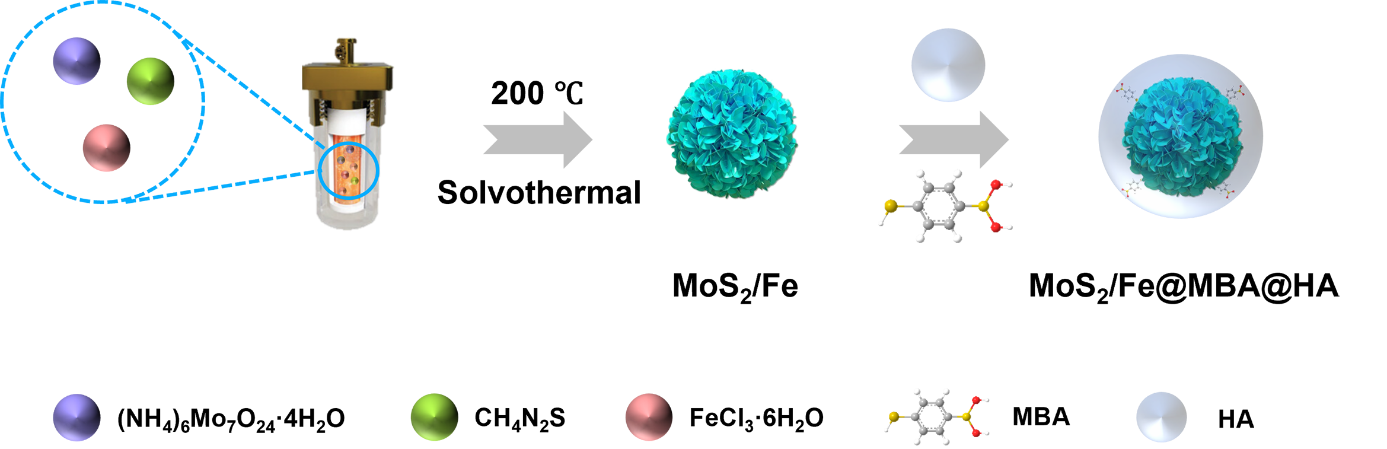


**Figure S1.** Schematic diagram of the process of MFBH preparation.


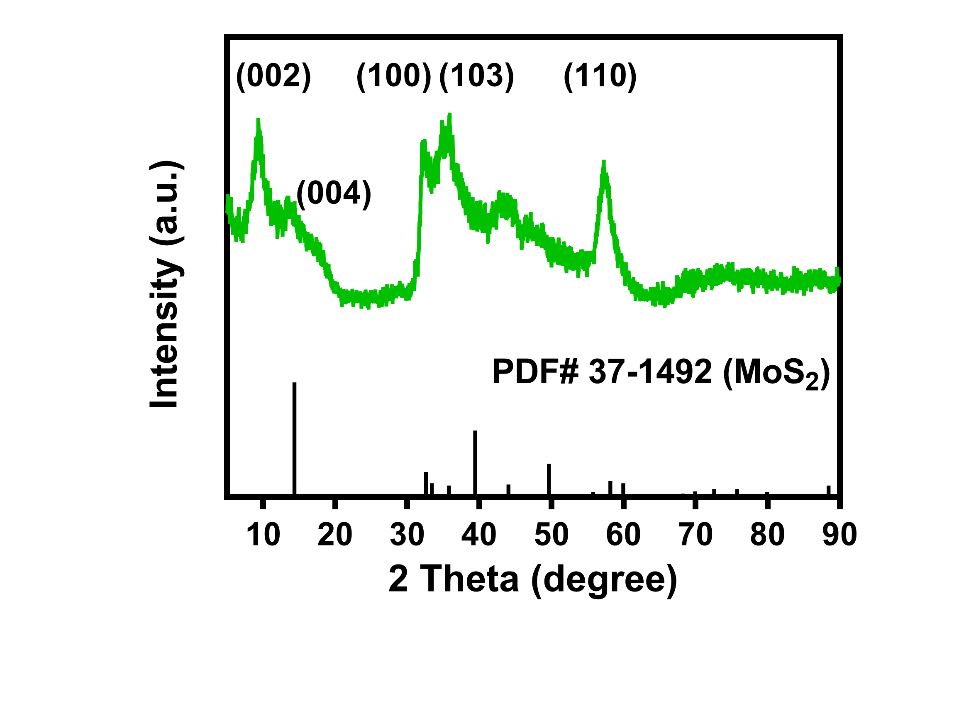


**Figure S2.** XRD pattern of MF nanoflowers.


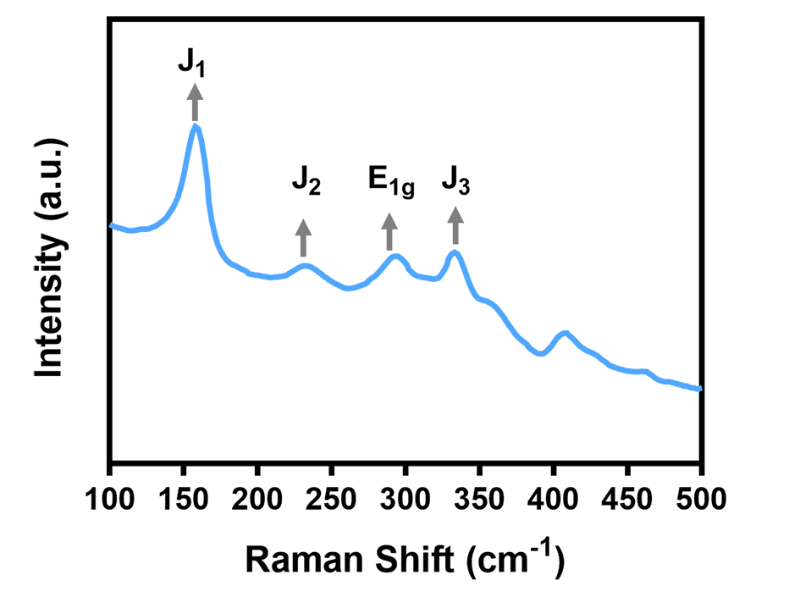


**Figure S3.** Reman spectrum of MF nanoflowers.


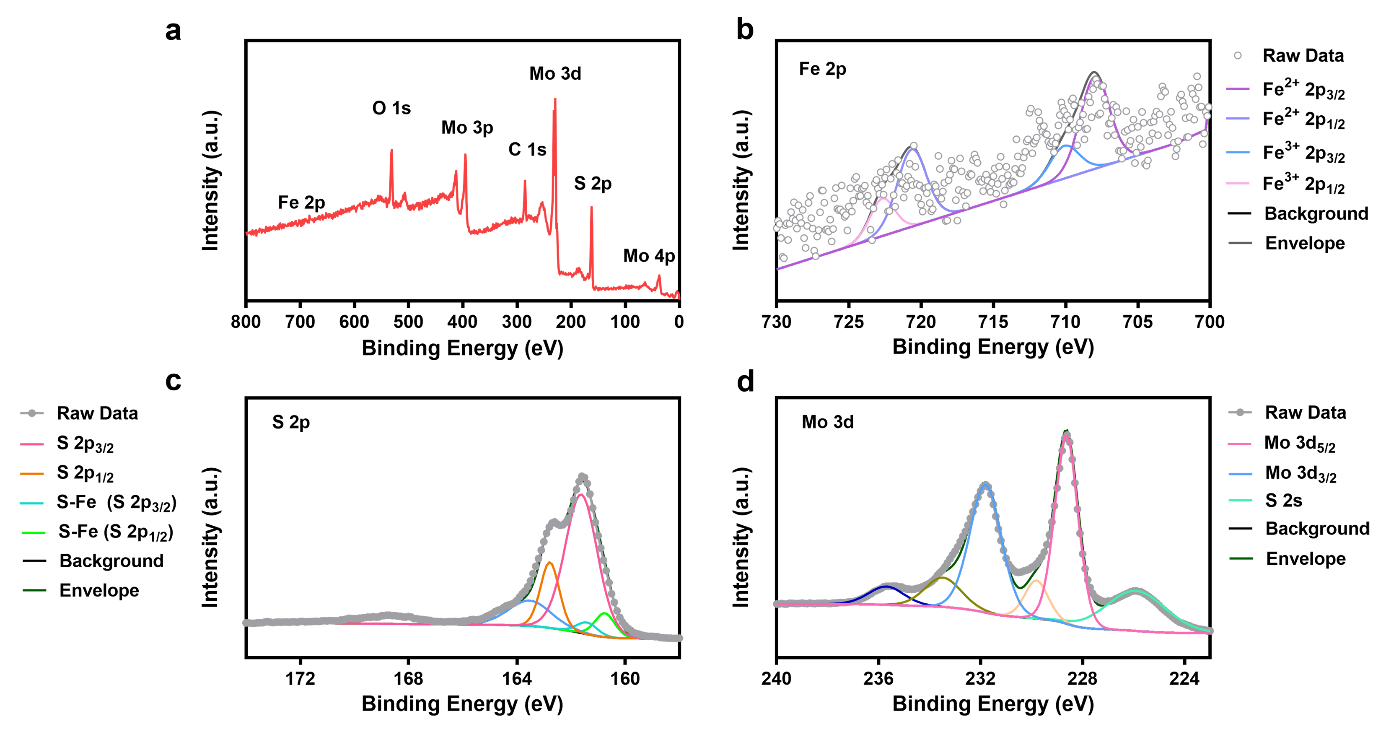


**Figure S4.** XPS spectrum of MF nanoflowers.


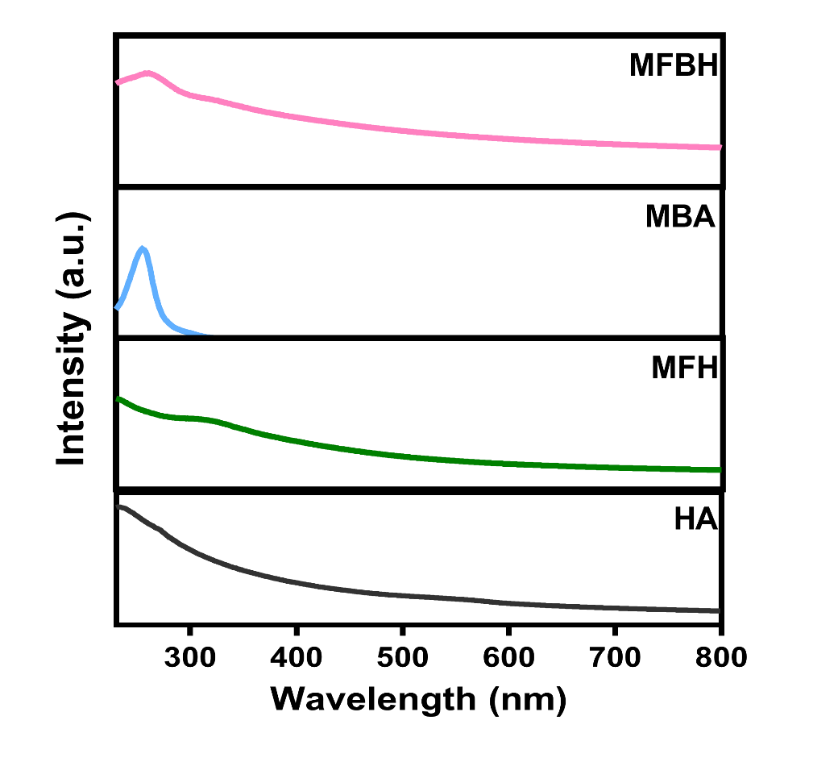


**Figure S5.** UV-vis spectrum of HA, MBA, MFH and MFBH.


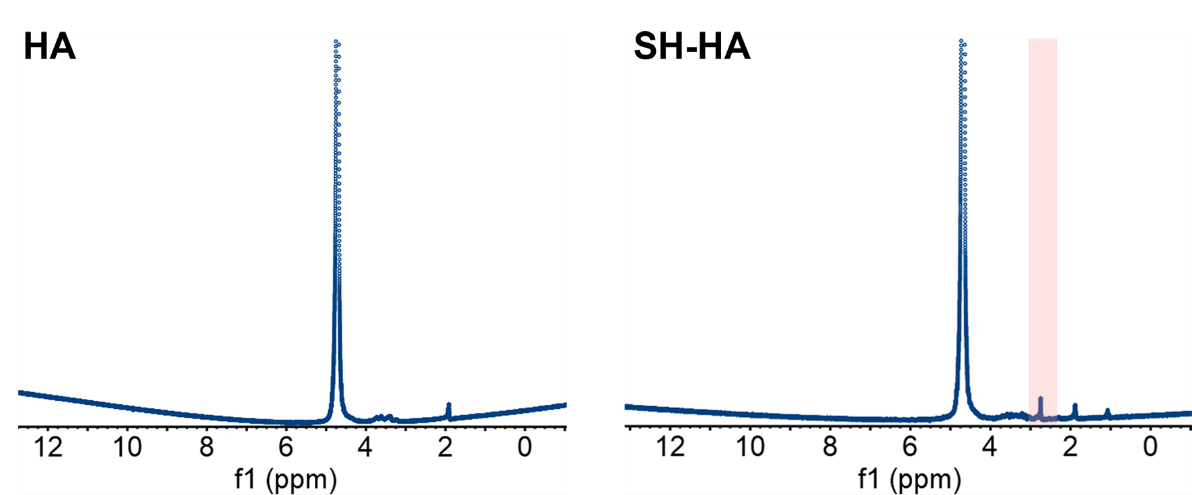


**Figure S6.** ^1^H NMR of HA and SH-HA.


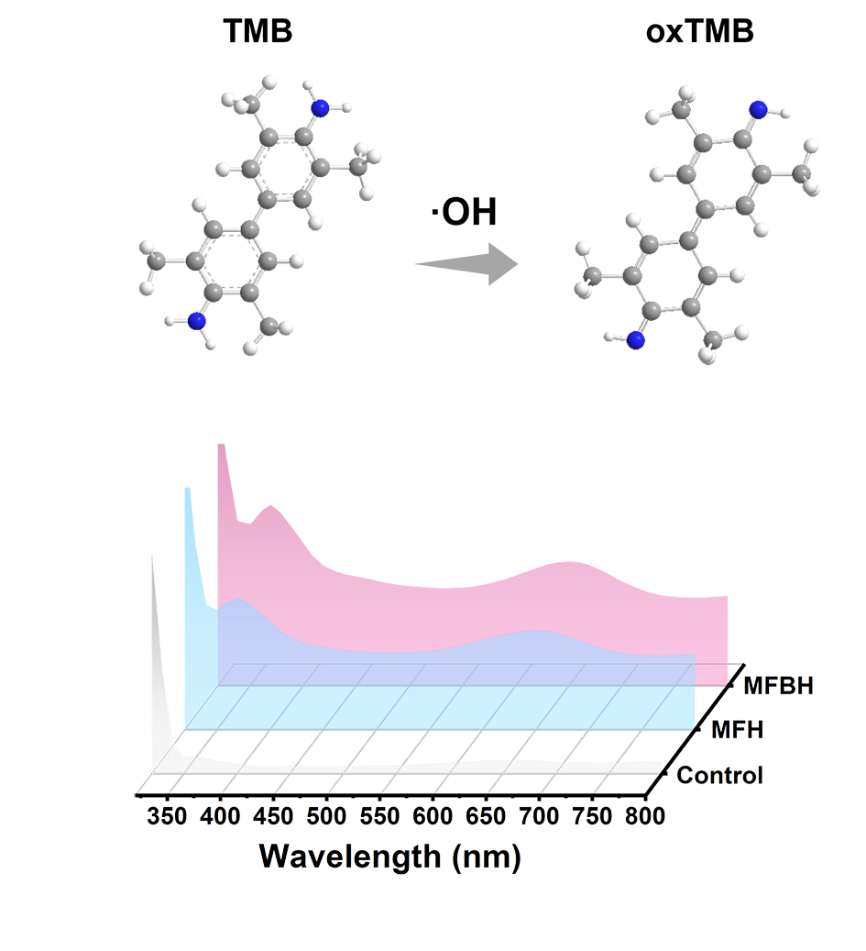


**Figure S7.** Mechanism of TMB oxidation and the UV-vis absorption spectrum of TMB treated with different artificial biocatalyst.


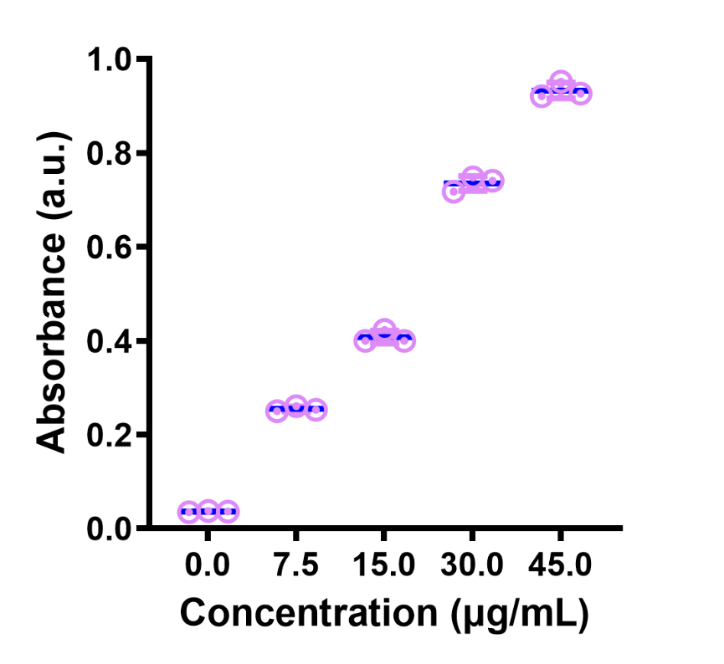


**Figure S8.** Concentration-dependent POD-like catalytic activity of MFBH. (Data are presented as mean ± SD. Signiﬁcance between two groups was calculated using one-way ANOVA and Tukey-Kramer multiple comparisons test. n = 3).


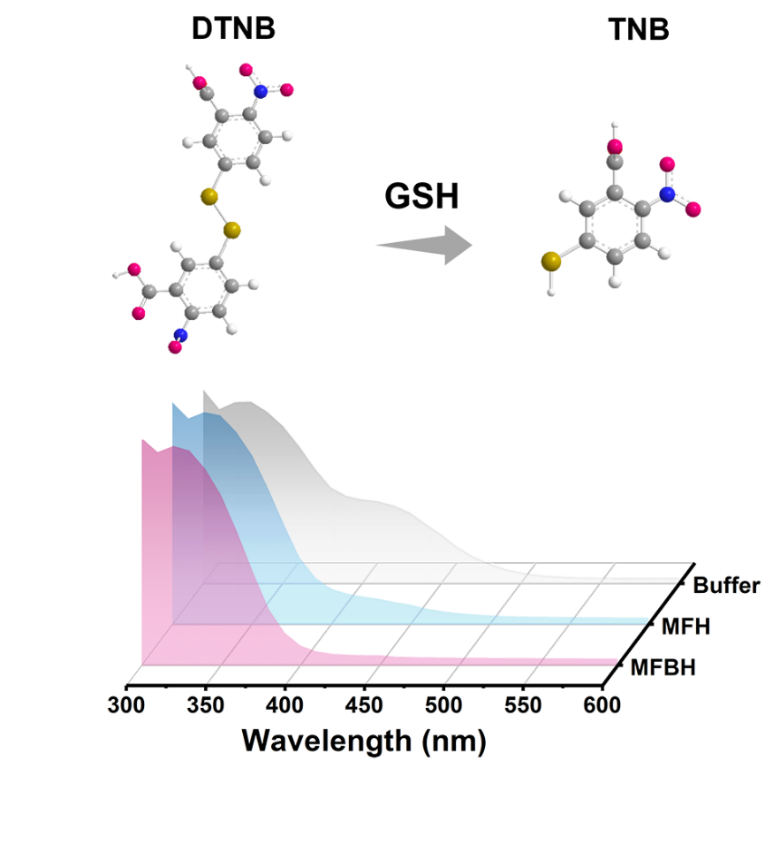


**Figure S9.** Principle of GSH detection and the UV-vis absorption spectrum of GSH treated with different artificial biocatalyst. DTNB: 5,5'-Dithiobis-(2-nitrobenzoic acid).


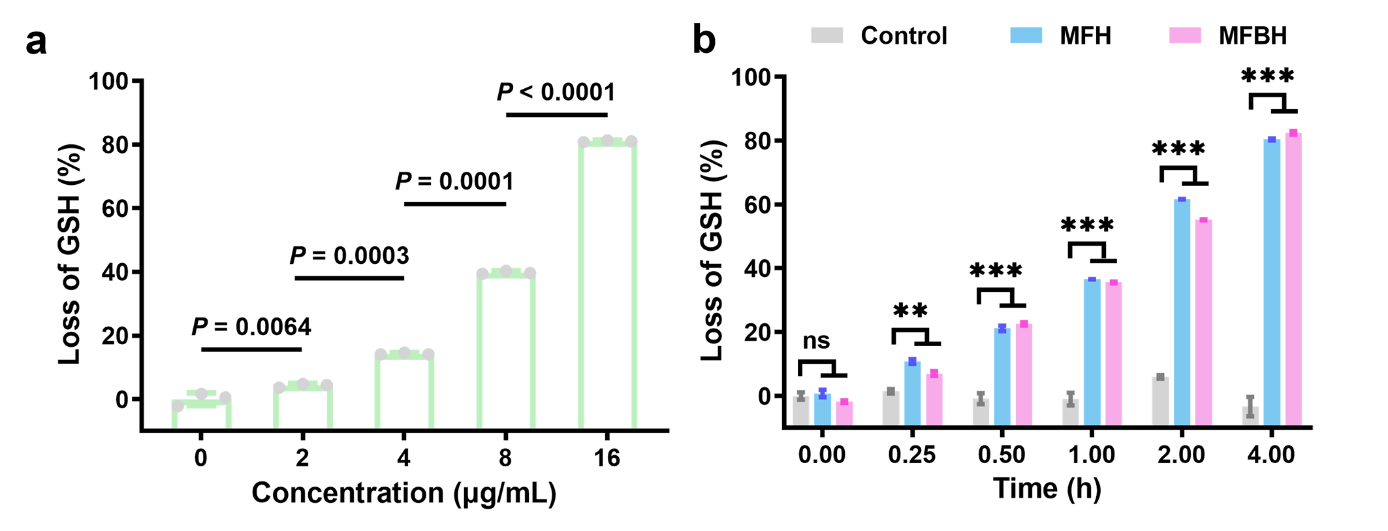


**Figure S10.** Concentration-dependent and time-dependent GSH-depleting activity of MFBH. (Data are presented as mean ± SD. Statistical significance was assessed using Student's t-tests. n = 3.)


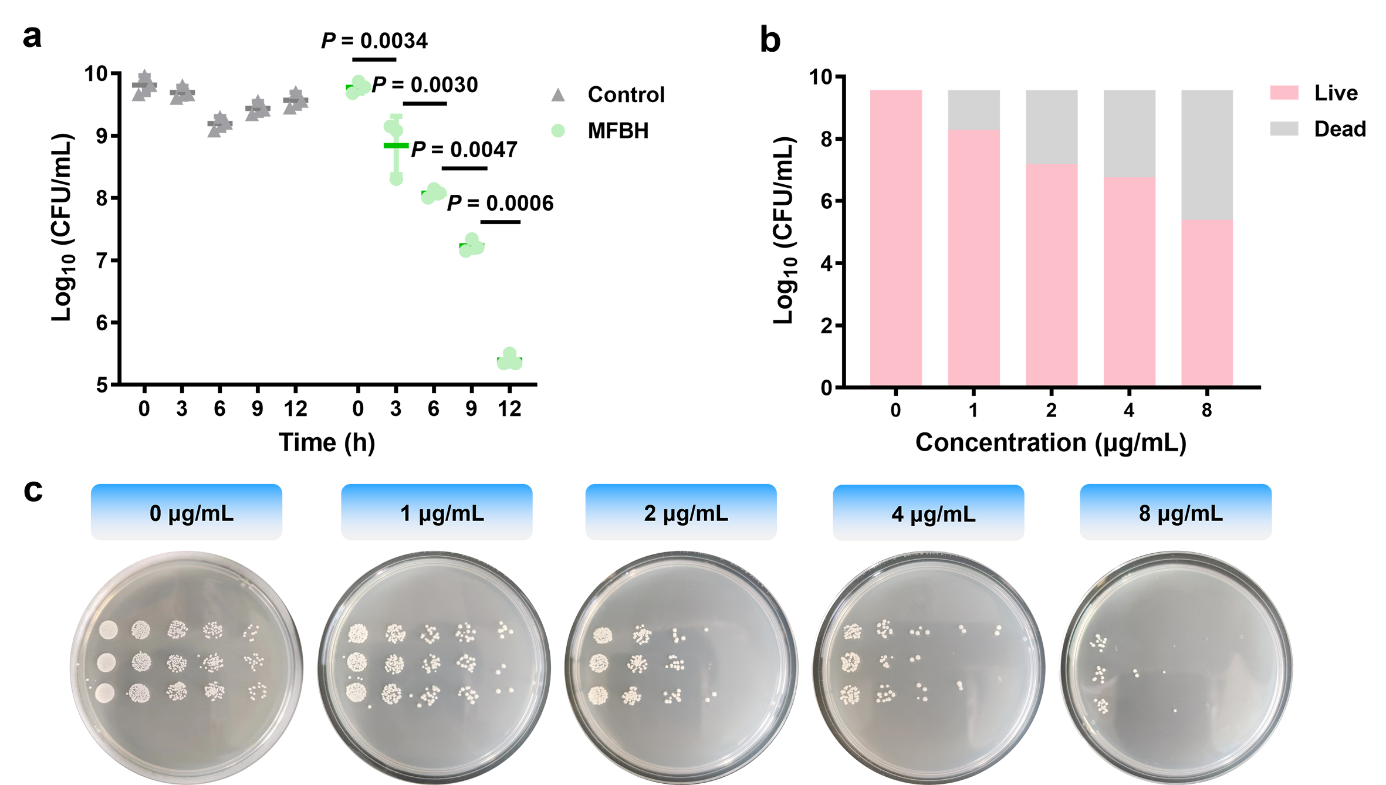


**Figure S11.** Antibacterial effect of MFBH. a) The number of colonies at different incubation times. b) The number of colonies after treatment with different concentrations of MFBH and c) the corresponding agar plates. (Data are presented as mean ± SD. Statistical significance was assessed using Student's t-tests. n = 3.)


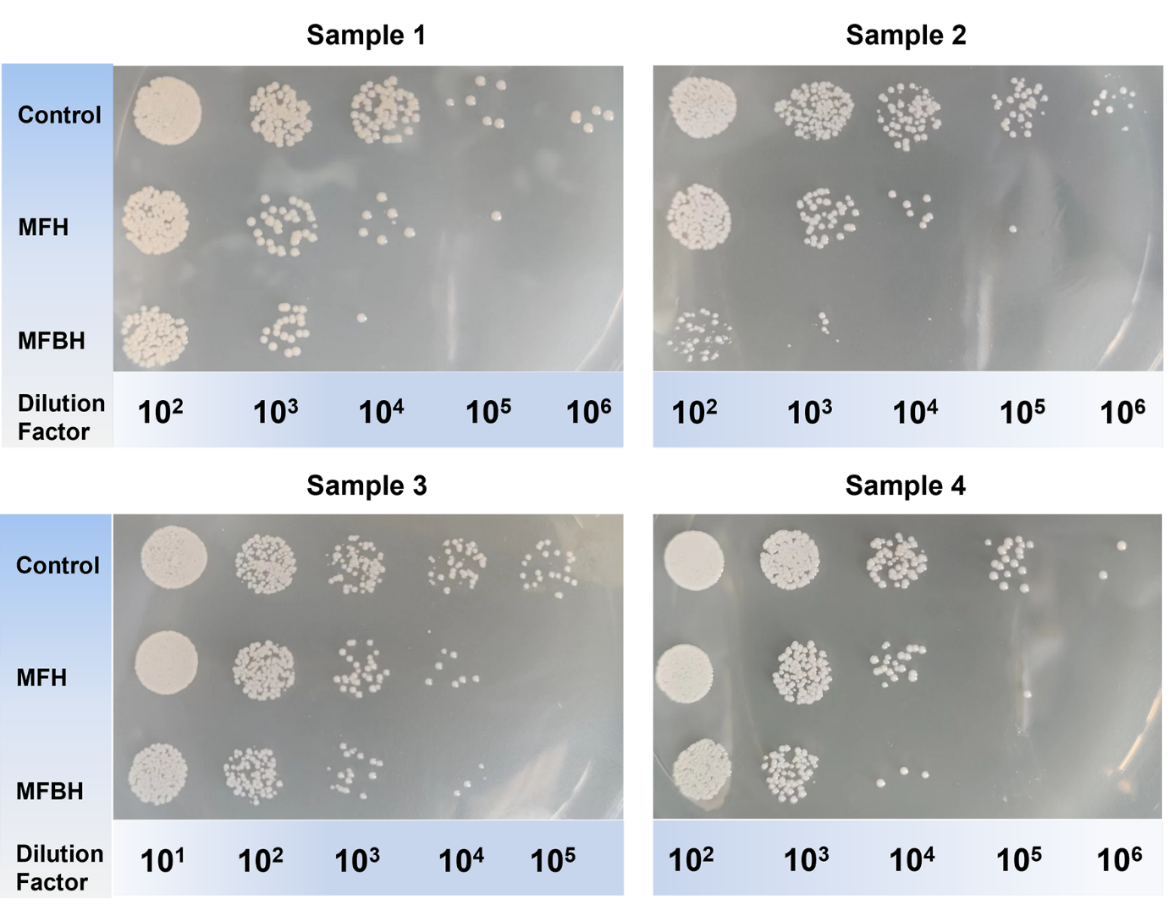


**Figure S12.** Antibacterial effect of MFBH against clinically isolated *S. aureus*.


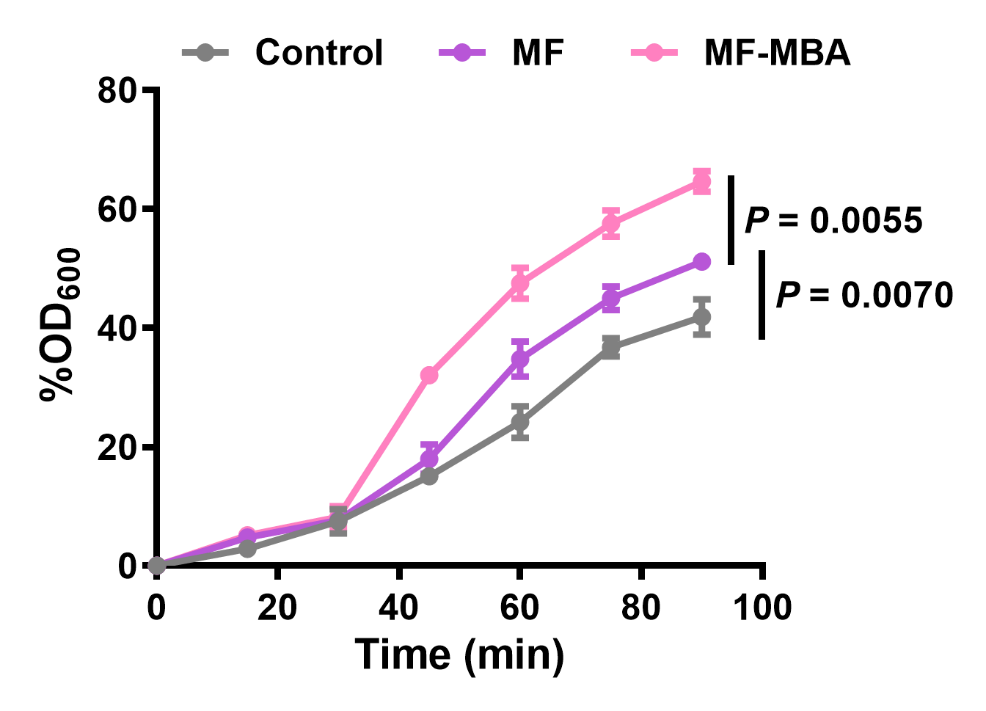


**Figure S13.** Changes in the %OD_600_ of *S. aureus* supernatants. (Data are presented as mean ± SD. Statistical significance was assessed using Student's t-tests. n = 3.)


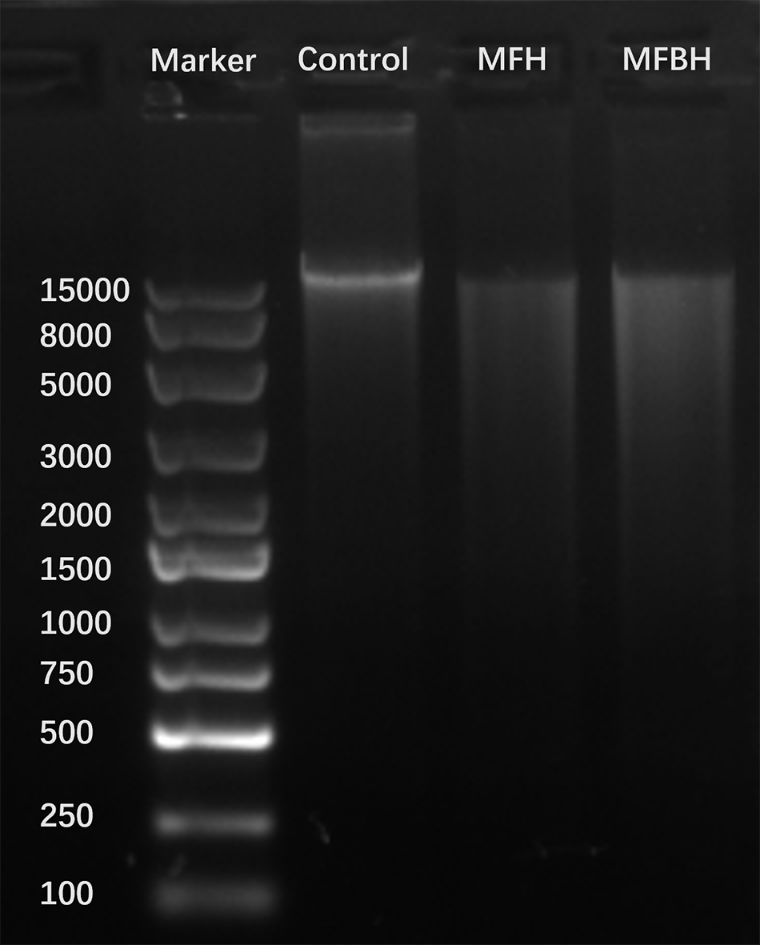


**Figure S14.** Agarose gel electrophoresis of bacterial DNA after different treatments.


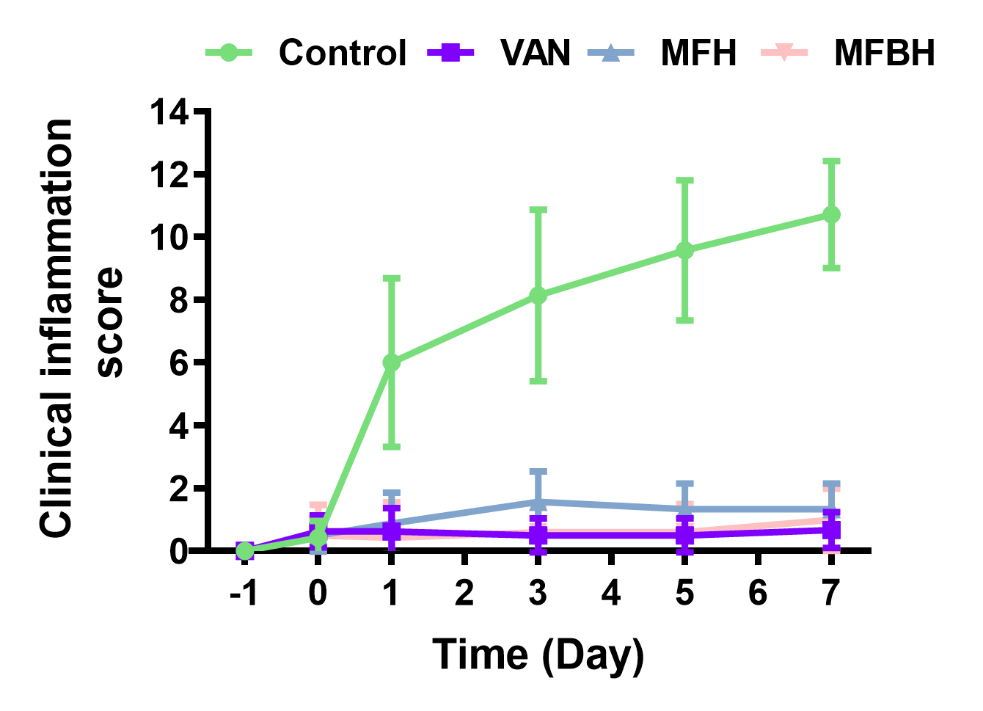


**Figure S15.** Clinical inflammation score of rat eyes on days -1, 0, 1, 3, 5 and 7. (Data are presented as mean ± SD. Statistical significance was assessed using Student's t-tests. n = 3.)


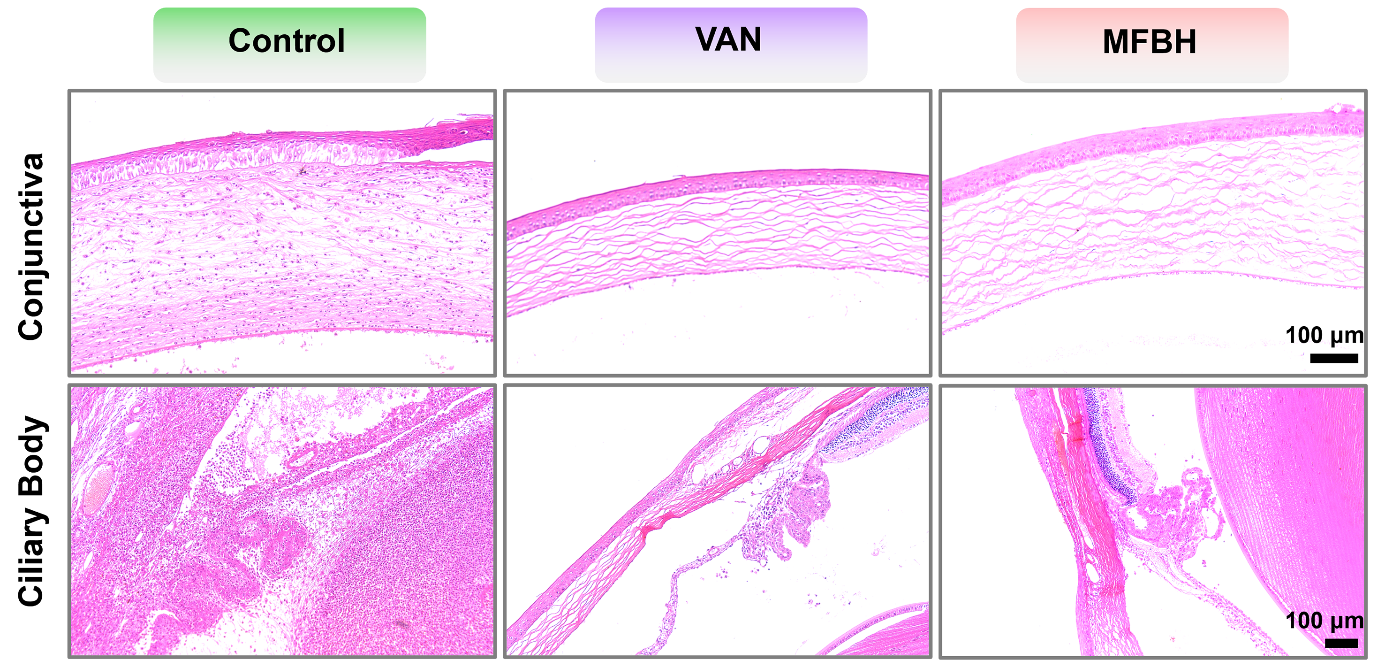


**Figure S16.** H&E staining of conjunctiva and ciliary body on day 7.


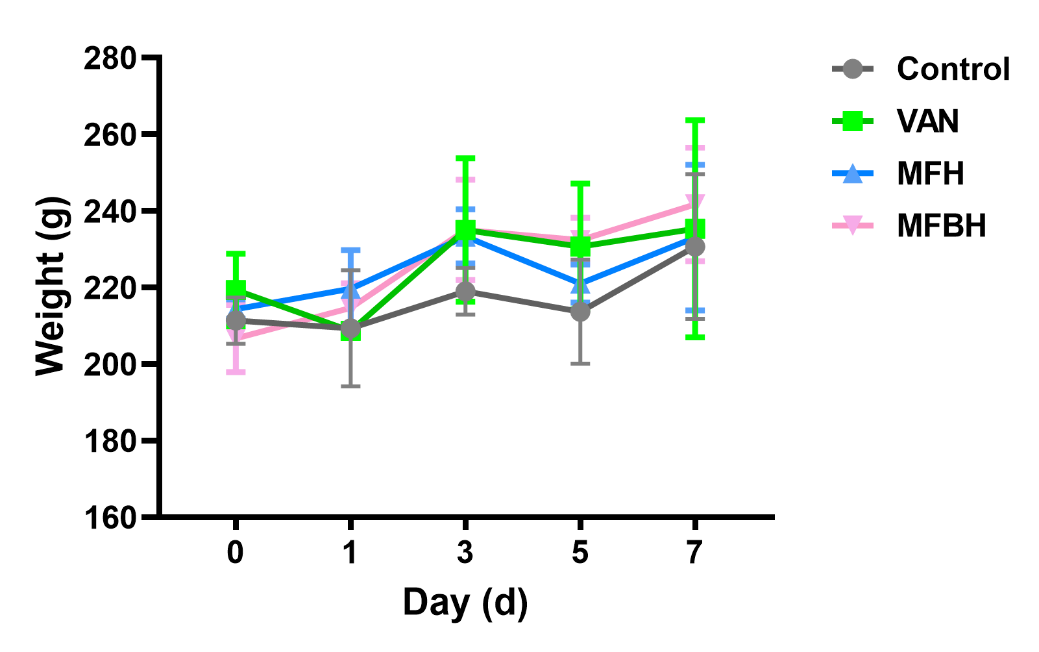


**Figure S17.** Changes in body weight of rats. (Data are presented as mean ± SD. Statistical significance was assessed using Student's t-tests. n = 3.)


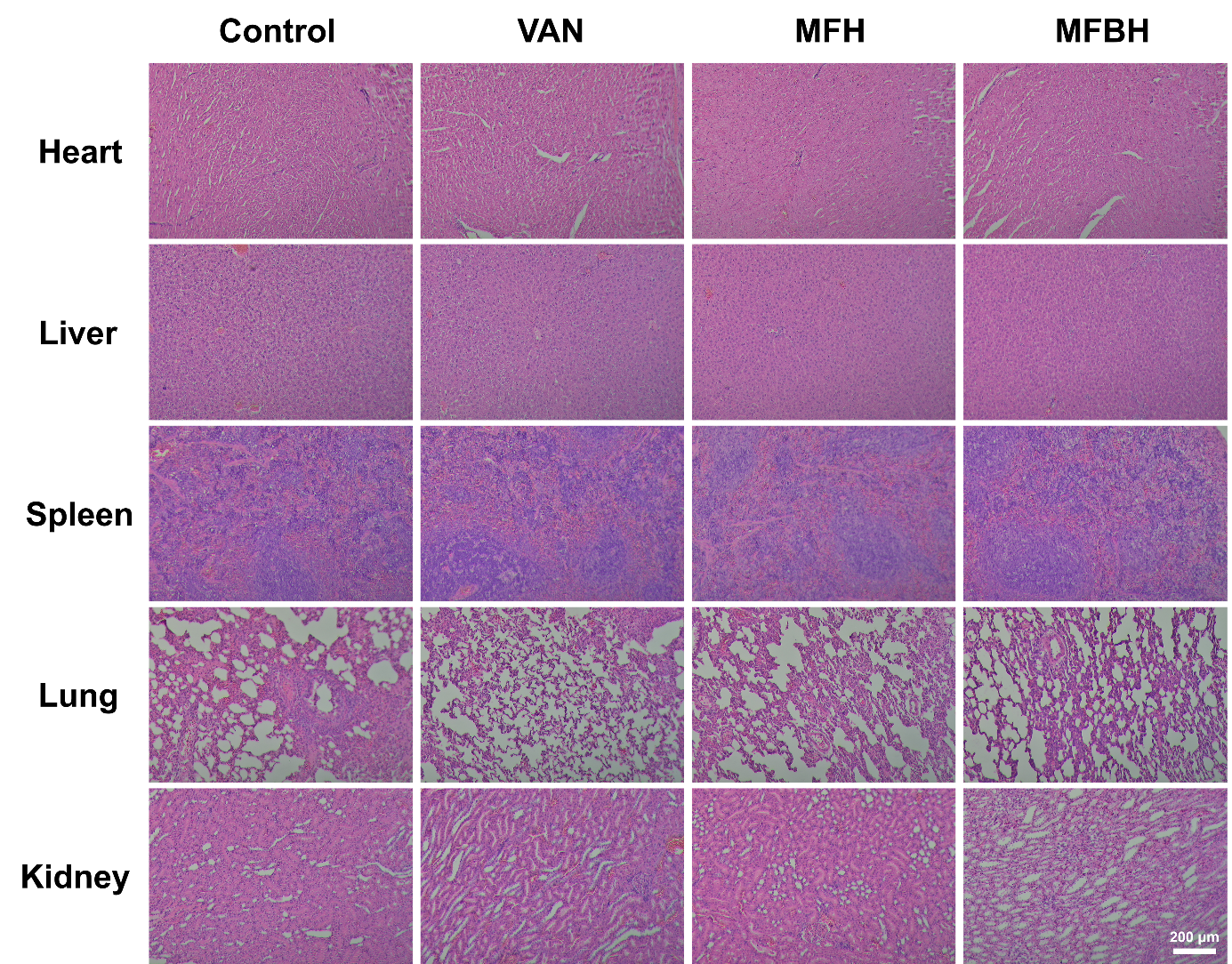


**Figure S18.** H&E staining of major organs, including heart, liver, spleen, lung and kidney, on day 7.


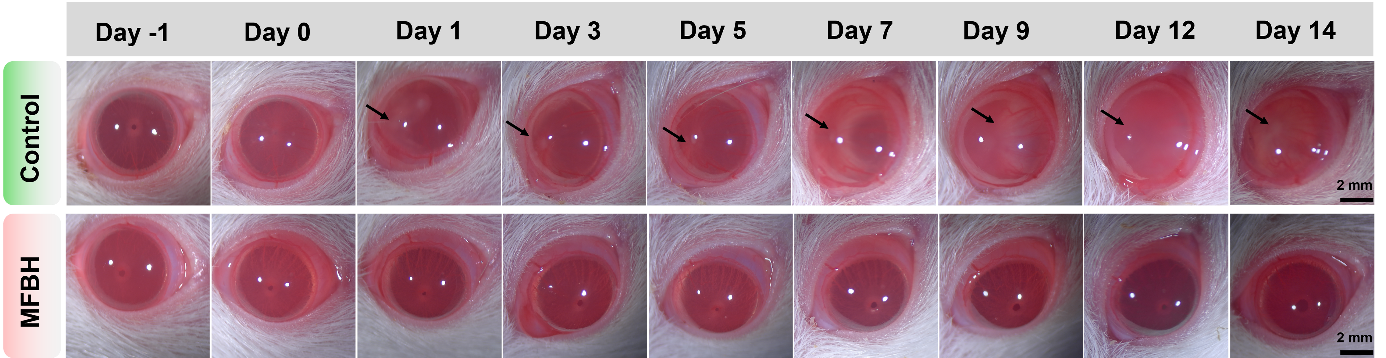


**Figure S19.** Representative photographs of rat eyes after administration on days -1, 0, 1, 3, 5, 7, 9, 12 and 14. MFBH was injected intravitreally on day 0. The black arrows indicate fibrinous exudations.


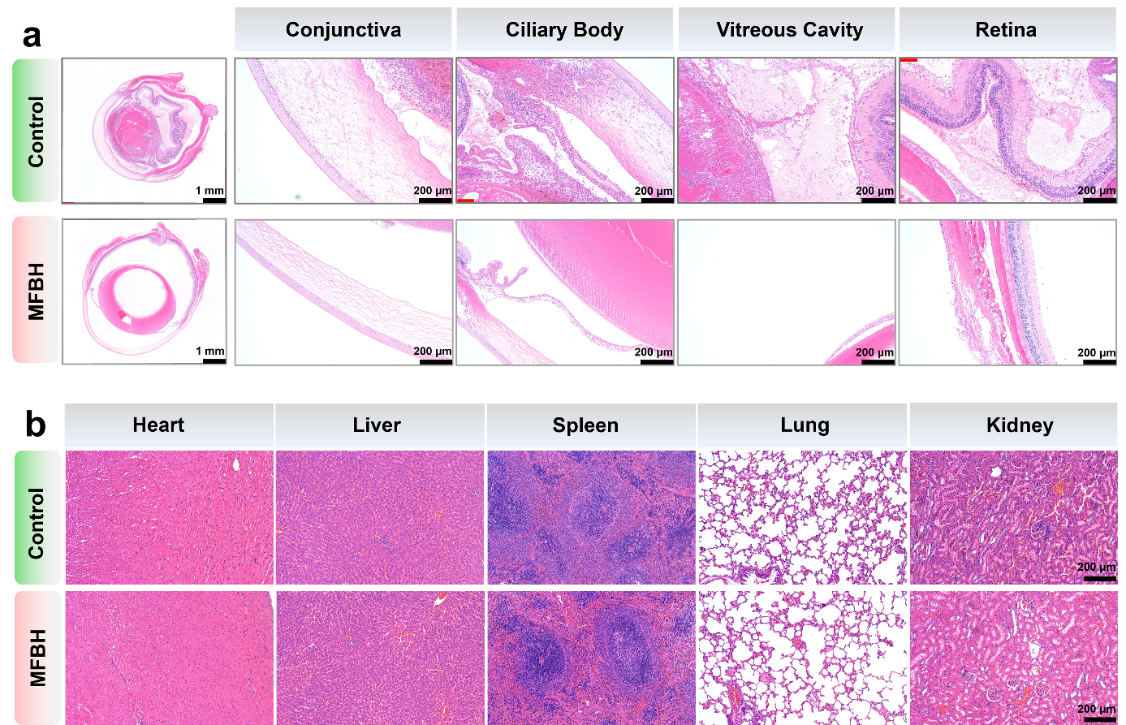


**Figure S20.** Pathological histology analysis. a) H&E staining of the conjunctiva, ciliary body, vitreous cavity and retina on day 14. b) H&E staining of major organs including heart, liver, spleen, lung and kidney on day 14.


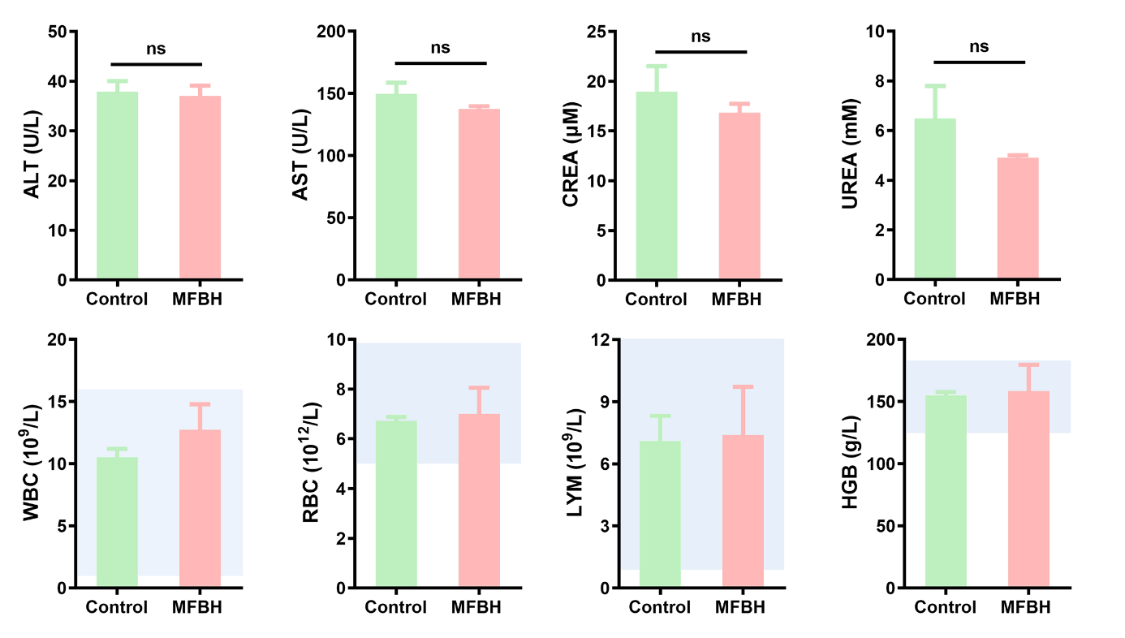


**Figure S21.** Hematology analysis of rats after different treatments including serum biochemistry (ALT, AST, CREA and UREA) and blood routine (WBC, RBC, LYM and HGB). ALT: alanine transferase; AST: aspartate transferase; CREA: creatinine; UREA: urea, WBC: white blood cells; RBC: red blood cells; LYM: lymphocytes; HGB: hemoglobin. The shaded areas represent the normal ranges. (Data are presented as mean ± SD. Statistical significance was assessed using Student's t-tests. n = 3.)


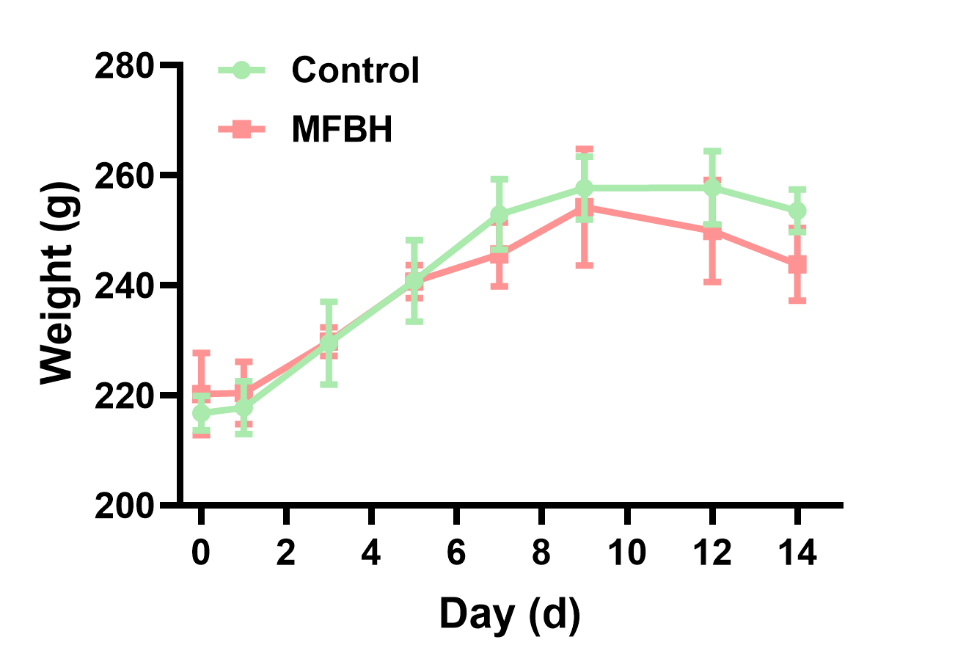


**Figure S22.** Changes in body weight of rats in the observation course of 14 days. (Data are presented as mean ± SD. n = 3.)


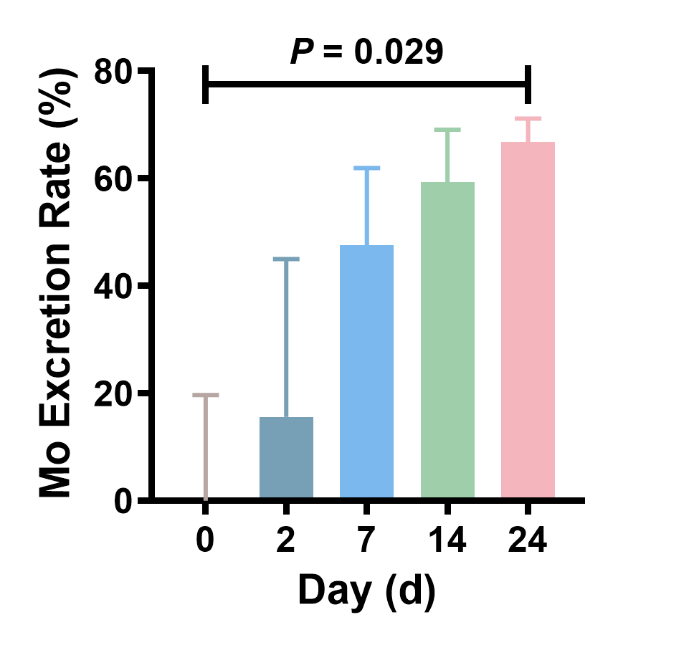


**Figure S23.** Mo levels within the eye detected by ICP-MS on days 0, 2, 7, 14 and 24 d after MFBH treatment. (Data are presented as mean ± SD. Statistical significance was assessed using Student's t-tests. n = 3.)


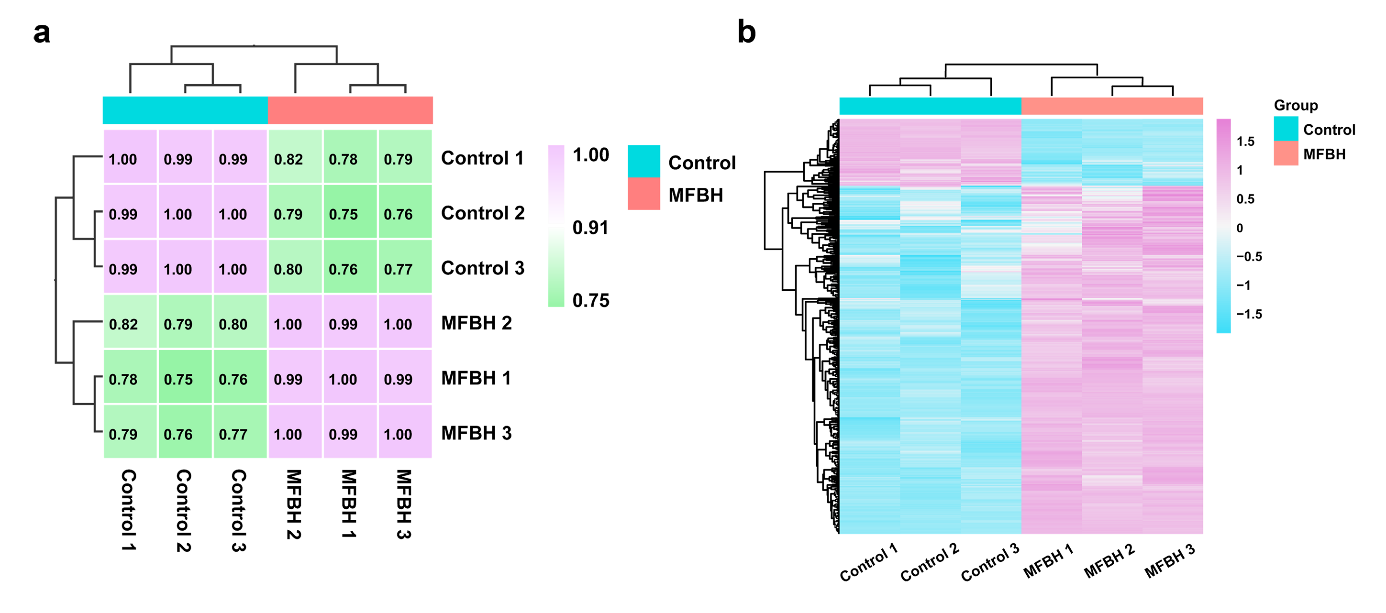


**Figure S24.** Transcriptomic changes of *S. aureus* treated with MFBH. a) Correlation analysis of all samples. b) Clustering heatmap of total diﬀerentially expressed genes.


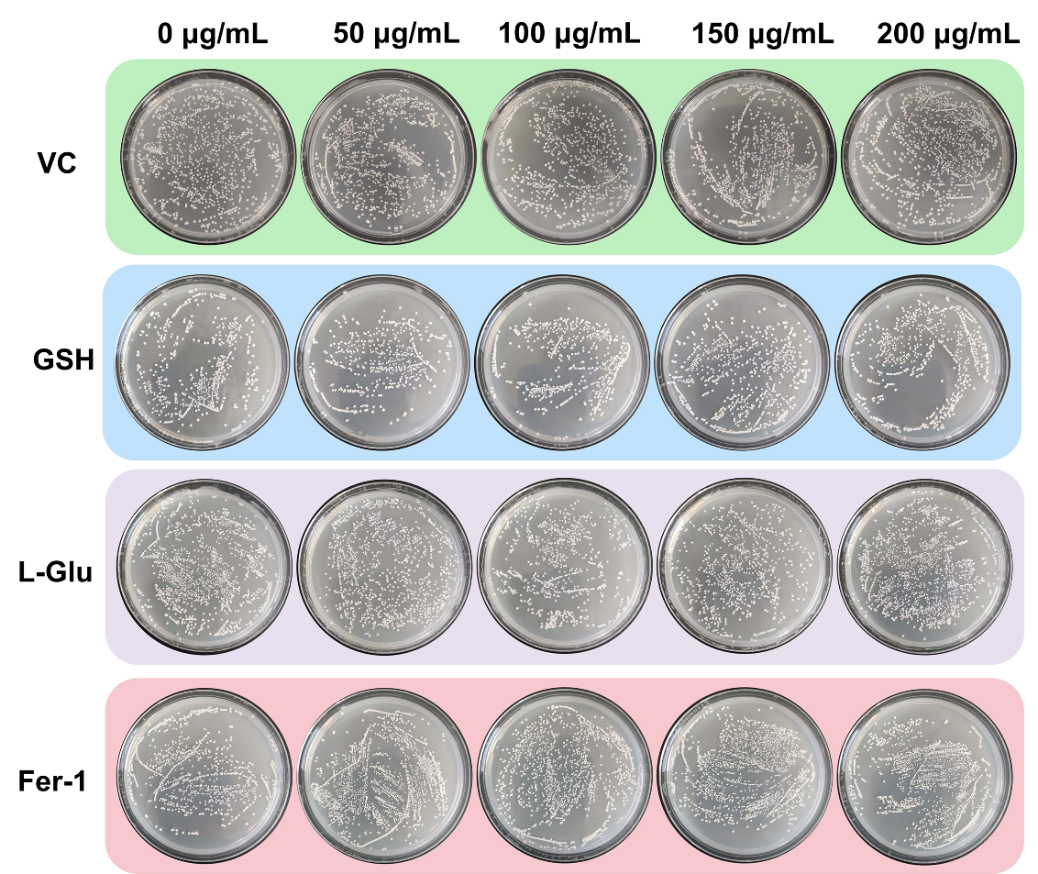


**Figure S25.** Agar plate images of *S.aureus* treated with different concentration of VC, GSH, L-Glu and Fer-1.


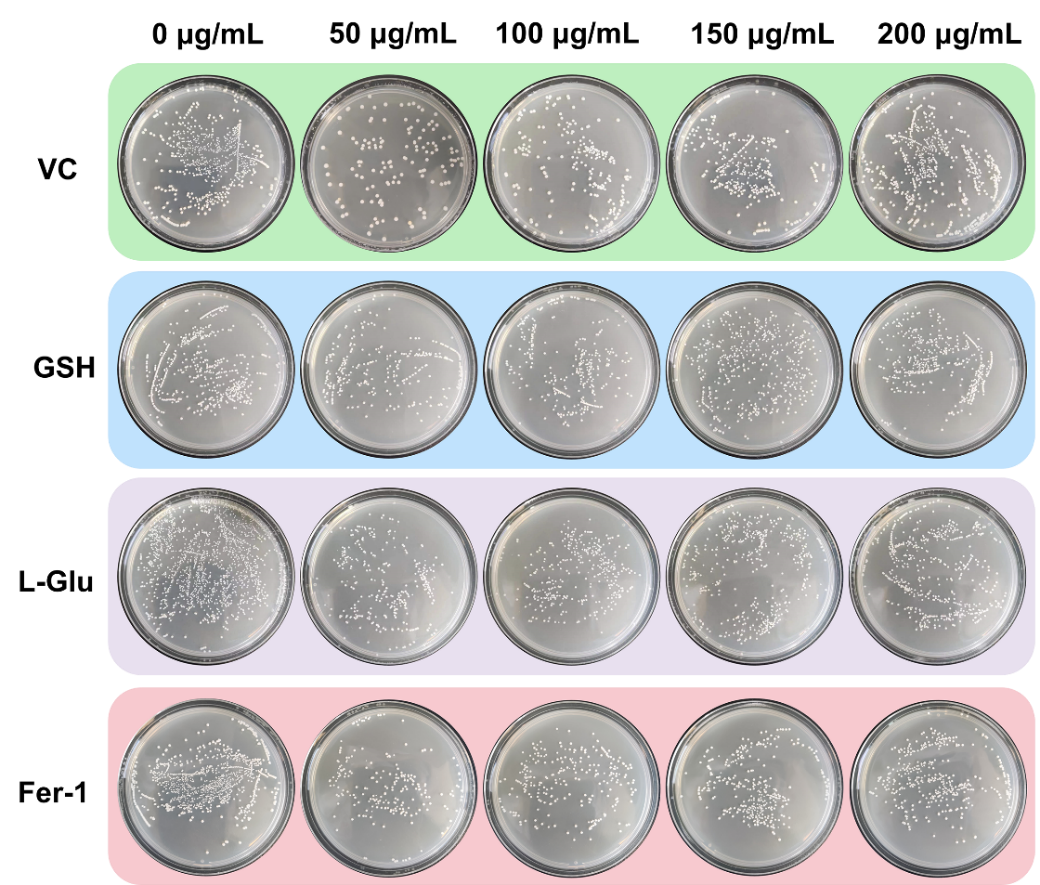


**Figure S26.** Agar plate images of *S.aureus* treated with MFBH and different concentration of VC, GSH, L-Glu and Fer-1.


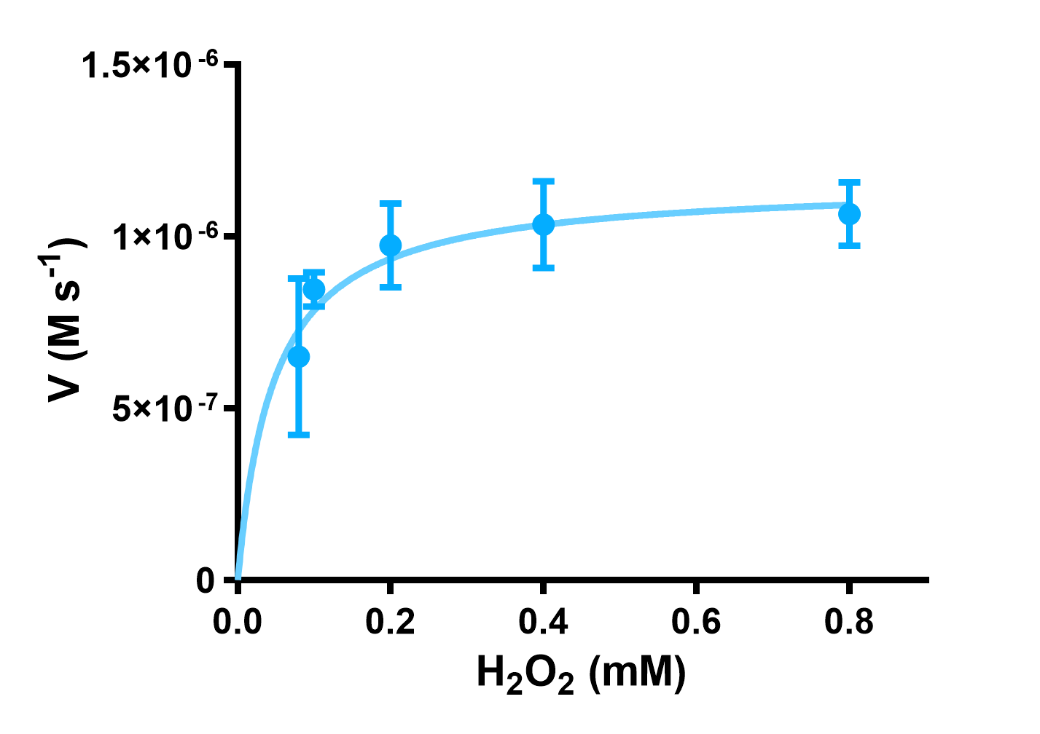


**Figure S27.** Steady-state kinetic assay of MF nanoflowers. (Data are presented as mean ± SD. n = 3.)


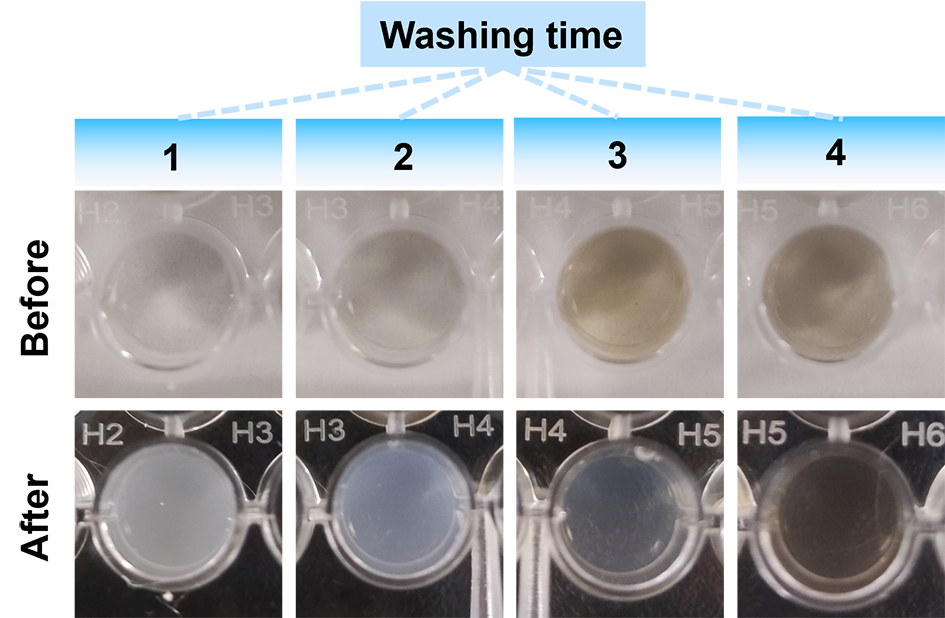


**Figure S28.** Representative photographs of washing solution of the MF nanoflower before and after the addition of silver nitrate solution.


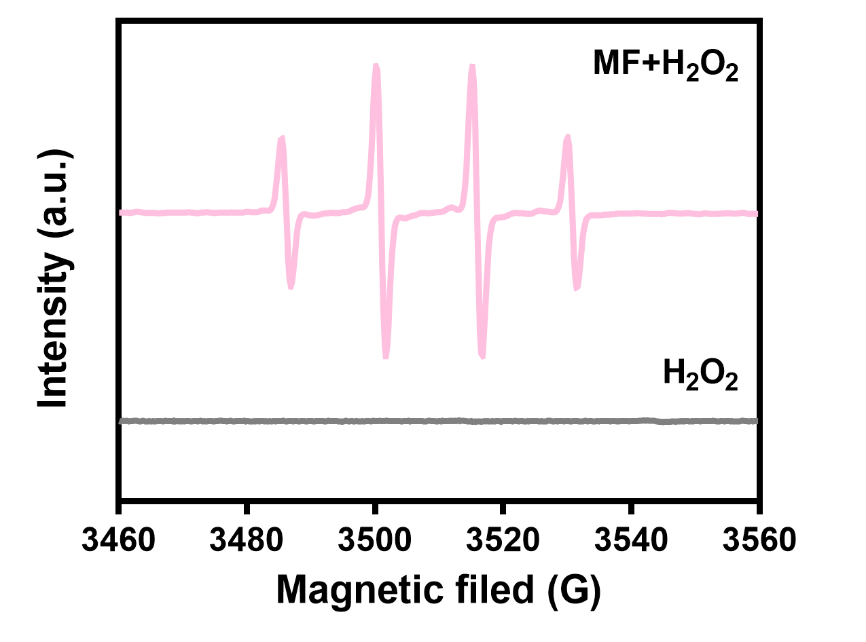


**Figure S29.** ESR spectrum of hydroxyl radical.


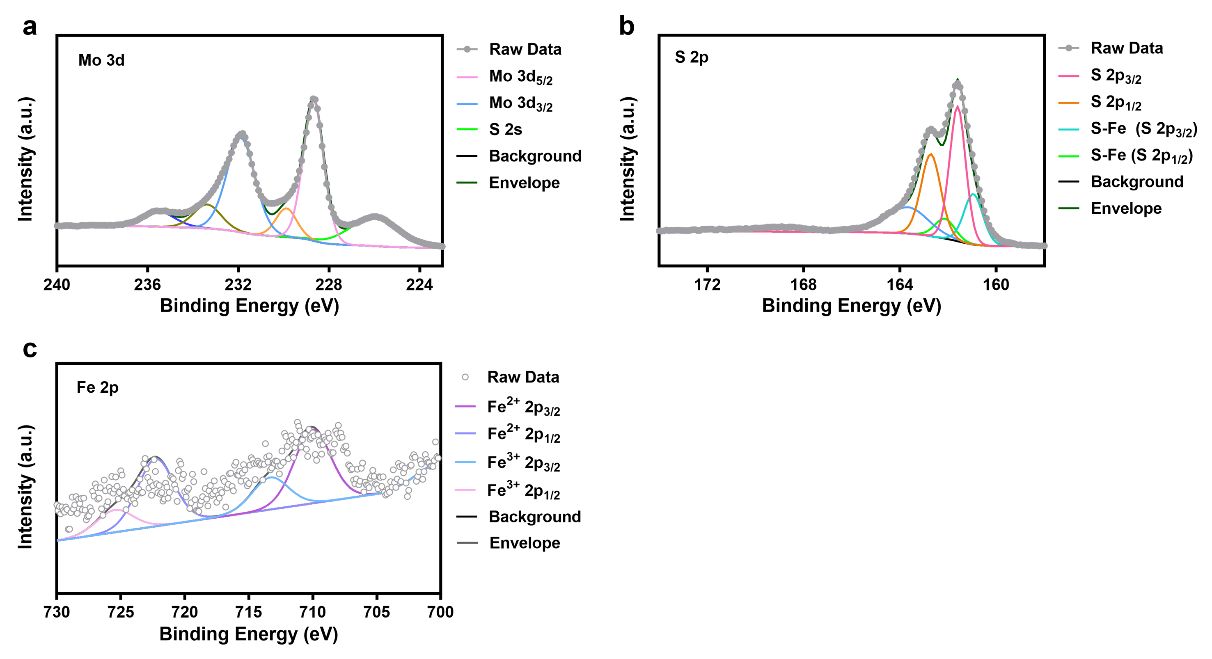


**Figure S30.** XPS spectrum of MF nanoflowers after treatment with H_2_O_2_.


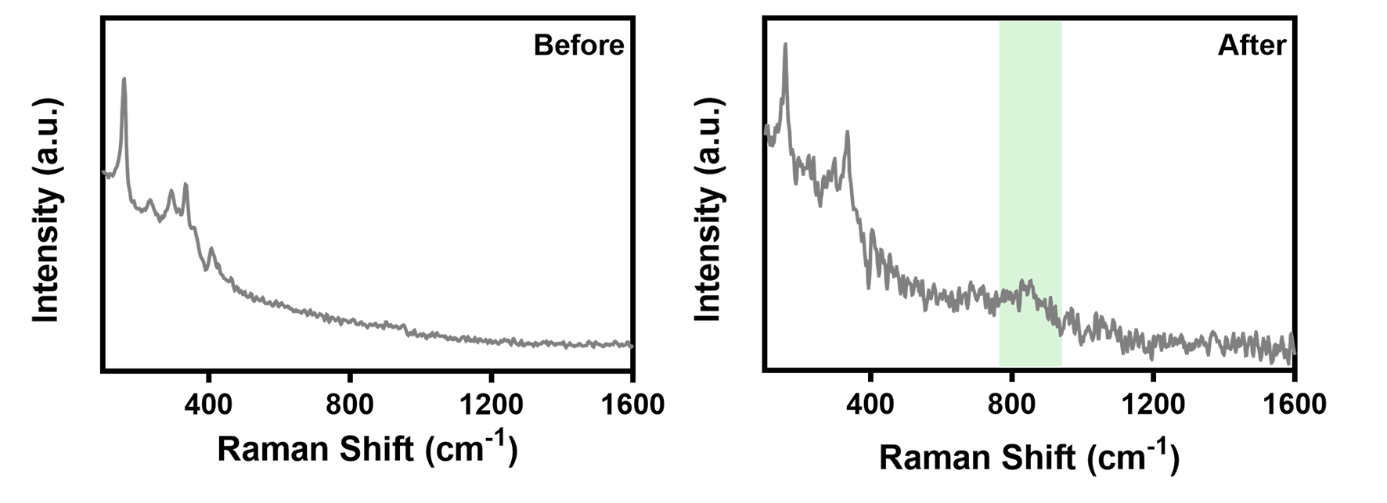


**Figure S31.** Reman spectrum of MF nanoflowers before and after treatment with H_2_O_2_.

**Table S1.** K_m_ values of different nanozymes.

| Nanozyme | K_m_ [mм] | References |
| --- | --- | --- |
| Iron-based single-atom nanozymes | 18.80 | [1] |
| Iron manganese silicate nanozyme | 49.80 | [2] |
| S-doped spatially asymmetric single-atom nanozymes | 0.45 | [3] |
| Calcium hexacyanoferrate nanoparticles | 4.71 | [4] |
| Protein-supported copper single-atom nanozyme | 0.48 | [5] |
| Single-platinum-atom | 2.94 | [6] |
| MOF@COF nanozyme | 0.45 | [7] |
| Na^+^/H_2_O co-intercalated MoO_3-x_ nanobelts | 2.41 | [8] |
| Zn/Mo dual single-atom catalyst supported on the macroscope aerogel | 40.32 | [9] |
| Zn_80_Co_20_-1000 | $\text{1.53×10}\text{-9}$ | [10] |
| MF nanoflower | $\text{3.2×10}\text{-4}$ | This work |

**References**

[1] R. Zhang, B. Xue, Y. Tao, H. Zhao, Z. Zhang, X. Wang, X. Zhou, B. Jiang, Z. Yang, X. Yan, K. Fan. Edge-Site Engineering of Defective Fe-N_4_ Nanozymes with Boosted Catalase-Like Performance for Retinal Vasculopathies. *Adv. Mater.* **2022,** *34*, 2205324.

[2] B. Xu, Y. Cui, W. Wang, S. Li, C. Lyu, S. Wang, W. Bao, H. Wang, M. Qin, Z. Liu, W. Wei, H. Liu. Immunomodulation-Enhanced Nanozyme-Based Tumor Catalytic Therapy. *Adv. Mater.* **2020,** *32*, 2003563.

[3] R. Niu, Y. Liu, B. Xu, R. Deng, S. Zhou, Y. Cao, W. Li, H. Zhang, H. Zheng, S. Song, Y. Wang, H. Zhang. Programmed Targeting Pyruvate Metabolism Therapy Amplified Single-Atom Nanozyme-Activated Pyroptosis for Immunotherapy. *Adv. Mater.* **2024,** *36*, 2312124.

[4] X. Shen, Z. Yang, X. Dai, W. Feng, P. Li, Y. Chen. Calcium Hexacyanoferrate Nanozyme Enhances Plant Stress Resistance by Oxidative Stress Alleviation and Heavy Metal Removal. *Adv. Mater.* **2024,** *36*, 2402745.

[5] X. Wang, Q. Chen, Y. Zhu, K. Wang, Y. Chang, X. Wu, W. Bao, T. Cao, H. Chen, Y. Zhang, H. Qin. *Signal Transduct. Target. Ther.* **2023,** *8*, 277.

[6] T. Ye, C. Chen, D. Wang, C. Huang, Z. Yan, Y. Chen, X. Jin, X. Wang, X. Ding, C. Shen. Protective Effects of Pt-N-C Single-Atom Nanozymes Against Myocardial Ischemia-Reperfusion Injury. *Nat. Commun.* **2024,** *15*, 1682.

[7] L. Zhang, Z. Liu, Q. Deng, Y. Sang, K. Dong, J. Ren, X. Qu. Nature-Inspired Construction of MOF@COF Nanozyme with Active Sites in Tailored Microenvironment and Pseudopodia-Like Surface for Enhanced Bacterial Inhibition. *Angew. Chem. Int. Ed.* **2021,** *60*, 3469.

[8] Z. Zhou, Y. Wang, F. Peng, F. Meng, J. Zha, L. Ma, Y. Du, N. Peng, L. Ma, Q. Zhang, L. Gu, W. Yin, Z. Gu, C. Tan. Intercalation-Activated Layered MoO_3_ Nanobelts as Biodegradable Nanozymes for Tumor-Specific Photo-Enhanced Catalytic Therapy. *Angew. Chem. Int. Ed.* **2022,** *61*, e202115939.

[9] C. Ma, Y. Xu, L. Wu, Q. Wang, J. Zheng, G. Ren, X. Wang, X. Gao, M. Zhou, M. Wang, H. Wei. Guided Synthesis of a Mo/Zn Dual Single-Atom Nanozyme with Synergistic Effect and Peroxidase-like Activity. *Angew. Chem. Int. Ed.* **2022**, e202116170.

[10] Y. Xing, L. Li, Y. Chen, L. Wang, S. Tang, X. Xie, S. Wang, J. Huang, K. Cai, J. Zhang. Flower-like Nanozyme with Highly Porous Carbon Matrix Induces Robust Oxidative Storm against Drug-Resistant Cancer. *ACS Nano* **2023,** *17*, 6731.
